# Advances in Computational Methodologies for Classification and Sub-Cellular Locality Prediction of Non-Coding RNAs

**DOI:** 10.3390/ijms22168719

**Published:** 2021-08-13

**Authors:** Muhammad Nabeel Asim, Muhammad Ali Ibrahim, Muhammad Imran Malik, Andreas Dengel, Sheraz Ahmed

**Affiliations:** 1 German Research Center for Artificial Intelligence (DFKI), 67663 Kaiserslautern, Germany; Muhammad_Ali.Ibrahim@dfki.de (M.A.I.); andreas.dengel@dfki.de (A.D.); sheraz.ahmed@dfki.de (S.A.); 2 Department of Computer Science, Technical University of Kaiserslautern, 67663 Kaiserslautern, Germany; 3 National Center for Artificial Intelligence (NCAI), National University of Sciences and Technology, Islamabad 44000, Pakistan; malik.imran@seecs.edu.pk; 4 School of Electrical Engineering & Computer Science, National University of Sciences and Technology, Islamabad 44000, Pakistan; 5 DeepReader GmbH, Trippstadter Str. 122, 67663 Kaiserslautern, Germany

**Keywords:** non-coding RNA classification, RNA sub-cellular localization, long non-coding RNA, small non-coding RNA, ncRNA, machine learning, deep learning, computational sequence analysis, benchmark performance, benchmark sequence analysis datasets

## Abstract

Apart from protein-coding Ribonucleic acids (RNAs), there exists a variety of non-coding RNAs (ncRNAs) which regulate complex cellular and molecular processes. High-throughput sequencing technologies and bioinformatics approaches have largely promoted the exploration of ncRNAs which revealed their crucial roles in gene regulation, miRNA binding, protein interactions, and splicing. Furthermore, ncRNAs are involved in the development of complicated diseases like cancer. Categorization of ncRNAs is essential to understand the mechanisms of diseases and to develop effective treatments. Sub-cellular localization information of ncRNAs demystifies diverse functionalities of ncRNAs. To date, several computational methodologies have been proposed to precisely identify the class as well as sub-cellular localization patterns of RNAs). This paper discusses different types of ncRNAs, reviews computational approaches proposed in the last 10 years to distinguish coding-RNA from ncRNA, to identify sub-types of ncRNAs such as piwi-associated RNA, micro RNA, long ncRNA, and circular RNA, and to determine sub-cellular localization of distinct ncRNAs and RNAs. Furthermore, it summarizes diverse ncRNA classification and sub-cellular localization determination datasets along with benchmark performance to aid the development and evaluation of novel computational methodologies. It identifies research gaps, heterogeneity, and challenges in the development of computational approaches for RNA sequence analysis. We consider that our expert analysis will assist Artificial Intelligence researchers with knowing state-of-the-art performance, model selection for various tasks on one platform, dominantly used sequence descriptors, neural architectures, and interpreting inter-species and intra-species performance deviation.

## 1. Introduction

As messenger ribonucleic acids (mRNAs) provide template for protein synthesis, mRNAs have been a major focus of Genomics research for a long period of time [1,2,3], whereas non-coding RNAs were widely considered the by-products of large transcription with very little biological importance [4]. Since the detection of transfer RNA and ribosomal RNA around 1950, a variety of RNA species have quite gradually emerged which exposed an unprecedented world of non-coding RNAs (ncRNAs) [5,6,7,8]. Large scale sequencing technologies as well as rich computational analysis have largely assisted with understanding the RNA world [9,10]. In the beginning of the 21st century, sequencing and computational analysis of the mouse [11] and human [12] genome indicated that 98% of junk DNA was possible to transcribed. However, it was not until the development of the Encyclopedia of DNA Elements (ENCODE) and Human Genome projects [13] in 2005 that revealed that almost 80% of human genome can be transcribed into various ncRNAs [14,15,16]. These projects have deeply explored ncRNAs in terms of identifying the sub-type of distinct ncRNAs and finding their biological roles. This progress has paved the way for the large scale identification of different ncRNAs such as long ncRNAs in different species (e.g., mammals) [17,18]. Afterwards, widespread application of deep advanced sequencing technologies enabled even more correct profiling of different ncRNAs [19,20].

Most recent literature reveals that ncRNAs act as key players in several biological, developmental, and physiological processes [21] as well as development and progression of complex diseases [21]. ncRNAs are also involved in gene expression regulation [22], RNA maturation [23], dosage compensation, genomic imprinting, and cell differentiation [8]. Contribution of ncRNAs in vital oncogenic processes such as differentiation, proliferation, migration, angiogenesis and apoptosis have gained much attention as potential diagnostic and prognostic biomarkers in leukemia [24]. Furthermore, ncRNAs were discovered to be tumor suppressors, oncogenic drivers in different cancer types [25], and strongly linked to the development of Alzheimer’s and cardiovascular diseases [26,27]. For plants, ncRNAs are considered as important regulatory molecules responsible for stress responses [28].

**Figure 1:**
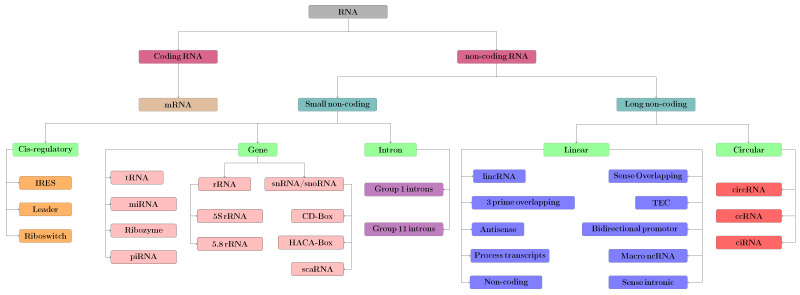
Hierarchical representation of RNA classes.

Based on cellular functionality, variation in sequence length, unique structure, and physical and chemical properties [29], ncRNAs can be segregated into different sub-classes, a taxonomy of which is depicted in Figure 1. Accurate discrimination of ncRNAs from coding RNAs and identification of their sub-types can lay the foundation for demystifying the core function and biological roles of ncRNAs, their involvement in suppressing the mechanism [30] underlying complex human diseases [31,32] or developing effective treatments and optimizing therapeutics [33,34]. Core functionality of biomolecules primarily relies on their distribution in various cellular compartments. The cellular role of particular RNA molecule can be studied using its localization information. Sub-cellular location information can decode the mechanisms of guiding post-transcriptional gene expression regulation ranging from epigenetic reprogramming all the way to post-transcriptional regulation [35,36] and directing RNA modification [37,38,39].

Due to the crucial roles of ncRNA in diverse cellular processes, disease development, and the potential to act as biomarkers, several experimental approaches have been developed to accurately identify ncRNA sub-type and sub-cellular localization. Among many experimental approaches, chemical and enzymatic RNA sequencing, effective parallel cloning to distinct ncRNAs using dedicated microarray analysis, genomic SELEX, and cDNA libraries are the most renowned ones [40]. Classification and sub-cellular location prediction of ncRNAs though experimental techniques is a costly and time-consuming approach. The influx of experimental data has given rise to comprehensive biological databases such as NONCODE [41], Rfam [42], circBase [43], miRbase [44], RNALocate [45], and the Ensembl database [46]. For instance, only the RNALocate meta-thesaurus has more than 190,000 entries for RNA sub-cellular localization along with experimental proofs such as 65 organisms (e.g., *Mucus musculus*, *Homo sapiens*, and *Saccharomyces cerevisiae*), 44 sub-cellular localizations (Endoplasmic reticulum, Nucleus, Cytoplasm, and Ribosome), and 9 RNA classes (e.g., miRNA, mRNA, and lncRNA) [45]. In addition, this database provides a large amount of annotated sequences for various classes of ncRNAs. Public availability of humongous data related to different sub-classes of ncRNA and their sub-cellular distribution has paved the way for the development of machine and deep learning approaches. Taking advantage of public databases, to date, several machine and deep learning based methodologies have been proposed to discriminate ncRNA from protein-coding transcripts, identify its sub-type, and predict their sub-cellular localization.

A variety of sequence and structure related features have been used by diverse uniquely designed computational predictors to accurately segregate ncRNAs from protein-coding transcripts, identify ncRNA sub-type, and infer their sub-cellular localization patterns using datasets of multiple species. The focus of this study is to shed light on distinct kinds of ncRNAs, discuss their biological importance, review machine and deep learning approaches proposed over the time to identify the sub-type of ncRNAs, and to predict their sub-cellular localization. It facilitates an interactive summary of benchmark datasets developed to evaluate the integrity of computational approaches for various tasks. We consider the facilitation of important elements in one platform such as benchmark performance of various genomic tasks, utilization trend with respect to sequence encoding, feature selection, and neural architectures, key research gaps, current challenges to effectively handle heterogeneity of different ncRNAs (e.g., sequence length, compositional variation), factors responsible for creating a bias towards residue distribution and high false positive rate will open a new horizon for the development of more robust computational approaches for diverse tasks. The contributions of this study can be summarized as:

1. A bird’s eye view on biological significance of diverse ncRNA species, their involvement in a wide range of cellular processes, disease development, and potential to act as biomarkers;
2. Taking heterogeneity of ncRNAs in terms of sequence length, structure, physical, and chemical characteristics into account, discussing the importance of distinguishing ncRNAs from protein-coding transcripts as well as identifying its sub-type;
3. Shedding lights on the significance of ncRNA sub-cellular localization information in regard to understand the core functionality of ncRNAs and their involvement in different biological processes;
4. Reviewing the progress of Artificial Intelligence for distinct ncRNA sequence analysis tasks including distinguishing ncRNAs from protein-coding transcripts, identifying the sub-type of ncRNAs, and sub-cellular localization;
5. Performing a critical analysis of diverse computational approaches proposed for different ncRNA sequence analysis tasks at different levels such as feature representation, feature selection, classification, and cross-species evaluation;
6. An interactive yet in-depth descriptive analysis of benchmark datasets developed using public database for diverse ncRNA sequence analysis tasks.

## 2. RNA Classification

Ribonucleic acid (RNA), a compound molecule, is considered indispensable for a variety of biological tasks in regulation, expression, coding, and decoding of genes https://www.umassmed.edu/rti/biology/role-of-rna-in-biology/ (accessed on 1 April 2021). Generally, RNA is categorized into coding or non-coding (ncRNAs) [47]. ncRNAs have been recognized in a broad variety of distinct classes or families, which vary in function, and provide insights into biological regulatory mechanisms in diseases that play an increasingly important role in searching for new drug targets by utilizing the information contained in genomics [47]. Since, in the identification of drug targeting, the regulatory circuits of ncRNA depend on knowing its family, the interest in developing sophisticated methods for ncRNA classification has dramatically increased over this period of time [48].

Here, we briefly describe state-of-the-art machine and deep learning approaches proposed to distinguish non-coding RNAs from coding RNAs and to identify sub-type of ncRNAs.

### 2.1. Distinguishing Long Non-Coding RNA from Protein Coding RNA

Long non-coding RNA transcripts (greater than 200 bp) are quite similar to protein-coding transcripts in terms of structure, transcriptional, as well as post-transcriptional behavior, indicating the difficulty in distinguishing lncRNAs from protein coding RNAs. Considering that the low expression level of lncRNAs largely hampers their identification using gene expression analysis technologies, several computational predictive methodologies have been developed by acquiring important biological features from sequences [49,50,51,52,53,54,55,56,57,58,59,60,61,62,63,64].

Stadler et al. [53] proposed a classification methodology, namely RNAz, which utilized support vector machine (SVM) to classify RNA sequences into coding and ncRNA (lncRNA) class. At pre-processing stage, the authors performed an alignment of sequences and developed a set of discriminative features using valuable information about thermodynamic stability as well as the secondary structure of sequences. RNAfold was used to acquire a secondary structure of sequences [65]. They evaluated the integrity of RNAz on a ncRNA dataset containing the sequences of five different species including human, mouse, Fugu, rat, and zebrafish taken from the Rfam database, where RNAz achieved an accuracy of 75%. A similar ncRNA classification methodology, namely “CONC”, was proposed by Liu et al. [54]. CONC used a combination of different features including peptide length, amino acid composition, compositional entropy, and number of homologs from database searches, and predicted the percentage of exposed residues, alignment entropy, and secondary structure to feed the SVM classifier. CONC achieved the specificity of 95% on the Eukaryotic species dataset for the task of distinguishing lncRNAs from protein-coding transcripts.

Kong et al. [57] presented a support vector machine based approach, namely the Coding Potential Calculator (CPC). CPC [57] measured the protein coding capability of a transcript by leveraging six biological sequence features including open reading frame score, coverage, integrity, number of hits, frame score, and number of high-scoring segment pairs. Experimental results revealed that CPC successfully discriminated lncRNA transcripts from coding transcripts with great accuracy of 98.6%. In addition, it was quite efficient compared to existing approaches CONC [54]. CPC can be accessed as a web service http://cpc.cbi.pku.edu.cn (accessed on 1 April 2021) where sequence features’ details and transcript annotations were facilitated to end users. However, the CPC web server requires a tremendous amount of time to process given sequences, which makes it non-effective for large scale sequence analysis. Furthermore, as CPC is an alignment based approach, its performance significantly fluctuates on the account of sequences taken from different reference databases. Building on these downfalls, later, authors upgraded CPC to CPC2 [58] by extracting four intrinsic sequence features: open reading frame (ORF) length, integrity, isoelectric point, and Fickett score to feed the SVM model. Using more training data, CPC2 [58] achieved better accuracy and speed as compared to CPC [57]. CPC2 can be accessed as a web service at http://cpc2.gao-lab.org/ (accessed on 1 April 2021) where sequence features details and transcript annotations services are facilitated to end users.

In order to provide an accurate method for the categorization of short genome regions in terms of coding or lncRNA, Lin et al. [66] presented PhyloCSF [66]. PhyloCSF [66] performed a deep analysis of nucleotide sequence arrangement belonging to multiple species in order to decide whether it was more likely to depict a conserved coding region. The authors illustrated that PhyloCSF [66] categorization performance over Drosophila 12-species genome alignments superseded the performance of existing approaches.Sun et al. [51] developed an “iSeeRNA” tool to discriminate lncRNAs from protein-coding RNAs. iSeeRNA [51] utilized three different features (conservation, sequence nucleotide composition, and open reading frame) to encode RNA sequences which were later passed to an SVM classifier to predict RNA family. Performance of iSeeRNA [51] was evaluated on Human and Mouse species datasets taken from RefSeq metathesaurus [67]. Over two different species datasets (Human and Mouse), iSeeRNA [51] achieved the accuracy of 96%, 95% for long intergentic ncRNA (lincRNA) identification, 94%, and 93% for the identification of protein-coding transcripts (PCTs). Performance comparison with two existing computational predictors indicated that iSeeRNA [51] outperformed PhyloCSF [66] by 13% for lincRNAs identification, 3% for PCTs identification, and CPC [57] by 10% for PCT identification. Sun et al. [55] proposed a robust signature tool, namely Coding-Non-Coding Index (CNCI), for the categorization of coding and non-coding transcripts. CNCI [55] utilized nucleotide triplets adjoined to each other to discriminate coding and lncRNA sequences regardless of known annotations. CNCI [55] was effective in classifying non-complete transcripts along with sense–antisense base pairs. To assess the effectiveness of CNCI [55] in comparison with existing computational predictors, authors compared the performance of CNCI [55] with CPC [57] and PhyloCSF [66]. Performance assessment revealed that CNCI [55] attained the accuracy of 97% for Human and Mouse datasets, which outperformed other predictor performance by 10%. Furthermore, CNCI [55] effectiveness for sense–antisense pairs revealed that CNCI [55] achieved the mean accuracy of 98% for non-coding-and-coding pairs, 97% for non-coding non-coding pairs, and 87% for coding–coding pairs which was better than other predictors’ performance by 3% for non-coding-coding pairs and 5% for coding–coding pairs. As CNCI utilized a sliding window to extract adjacent nucleotide triplets, selecting optimal residue context is not a straightforward task for different species because a window of a small size may lose important information, whereas a large window would take a huge amount of time to process given sequences.

Another alignment free tool was presented by Zhang et al. [56] who called it a predictor of long non-coding RNAs and messenger RNAs using an enhanced k-mer scheme (PLEK). The PLEK utilized a support vector machine along with an enhanced k-mer scheme specifically to discriminate lncRNA transcripts from messenger RNAs. Performance evaluation of PLEK over lncRNA and mRNA transcripts proved that PLEK achieved promising accuracy. In addition, PLEK was found to be suitable for great-scale transcriptomic data. PLEK evaluation over Human and Mouse datasets revealed that PLEK managed to achieve the accuracy of 96% for the Human dataset and over 90% on most cross-species test sets. Just like CNCI [55], PLEK also fails to handle the difference of nucleotides composition across different species and as a resut lacks the achievement of stable performance. Furthermore, Raghava et al. [50] presented SVM based methodology “LncRScan-SVM” to segregate lncRNAs from protein-coding RNAs. Using benchmark datasets of human and mouse species taken from the GENCODE database [68], LncRScan-SVM combined the features extracted from transcript sequence, gene structure, codon sequence as well as conservation to achieve the performance of 92% using an SVM classifier for the task of distinguishing lncRNAs from mRNAs.

Likewise, Schneider et al. [49] developed a “Longdist” approach which utilized open reading frame absolute length, relative length, and occurrences of K-mers selected using principal component analysis. To evaluate the effectiveness of the proposed approach, they utilized mouse, human and zebrafish datasets. Empirical evaluation indicated that the proposed approach obtained the top accuracy of 98% on the benchmark dataset, revealing the suitability of use for closely related species. Tong et al. [59] presented the coding potential prediction “CPPred” tool which utilized multiple sequence features including open reading frame length, integrity, coverage, hexamer score, Fickett score, pI, instability, gravy, and composition–transition–distribution features. Using the SVM classifier, they discriminated lncRNAs from protein-coding transcripts of five different species where top testing accuracy of 96% indicated the suitability of CCPred [59] for small sized transcripts. Wang et al. [52] developed another SVM based methodology “LGC” to identify lncRNAs belonging to a broad range of species. LGC [52] utilized a universal relationship that exists between open reading frame (ORF) length and guanine–cytosine (GC) content such that ORF length rises with GC content for those sequences which are rich in adenine and thymine, whereas it decreases for those sequences which are rich in guanine and cytosine. Due to unique paradigm, LGC [52] managed to identify lncRNAs with an accuracy of around 95% in cross-species setting using only one classification model.

Instead of SVM, few researchers utilized LR to accurately identify lncRNAs. For instance, Wang et al. [60] proposed an alignment free Coding Potential Assessment Tool (CPAT) to quickly discover coding and lncRNA transcripts from a huge collection of candidates using four sequence features. CPAT achieved top accuracy of 96% on the benchmark dataset which outshined baseline alignment based classification approaches by a significant margin. In addition, it was four times faster than CPC [57], and Phylo Codon Substitution Frequencies (PhyloCSF [66]) which enabled the users to process a huge collection of transcripts in no time. A minor downfall of CPAT is the selection of cutoff threshold which differs across different species; therefore, users have to find optimal cutoff for sequences of certain species in order to effectively identify lncRNA transcripts. Zhang et al. [61] developed another LR based methodology “LncScore” based on 11 different features including hexamer score, open reading frame length, coverage, hexamer score distance, maximum coding subsequences, and Fickett score belonging to three different feature groups. Using Human, Mouse, and other cross species datasets (fly, zebrafish, *Caenorhabditis elegans*, sheep, and rat), LncScore [61] achieved better performance as compared to existing predictors CPAT [60], PLEK [56], and CNCI [55]. LncScore achieved the top accuracy of 89% on partial length Human and Mouse testing datasets, 95% and 96% on full length Human and Mouse species datasets, outperforming existing predictors by 5% and 10% over aforementioned species, respectively.

Turning towards the methodologies based on decision tree, Achawanantakun et al. [62] developed “LncRNA-ID” methodology which extracted important features from three different segments including open reading frame, protein conservation, and ribosome interaction. To develop meta-classifiers based on multiple random forest, down-sampling was used to construct bootstrap samples. Using majority voting, LncRNA-ID [62] discriminated lncRNAs from protein-coding transcripts with the top accuracy of 96% on the benchmark dataset. Pian et al. [69] proposed another random forest based predictive methodology “LncRNApred” for accurate differentiation of lncRNAs from protein-coding transcripts. To evaluate the integrity of LncRNApred [69], they constructed the coding and non-coding transcripts dataset using UCSC [70] and NONCODE [41] databases. LncRNApred [69] used the self organizing feature map approach to learn rich representation of sequences which was passed to the meta-classifier for final prediction. Experimental results indicated that LncRNApred attained the accuracy of 93% on the benchmark dataset.

Hu et al. [63] developed coding potential calculation methodology, namely “COME”, which discriminated lncRNAs from protein coding transcripts using multiple sequence features and random forest classifier. To prove the integrity of COME [63], authors compared the performance of COME with CNCI [55], CPAT [60], and PhyloCSF [66] methodologies using two test sets of human species. They additionally evaluated COME on cross-species datasets as well including *M. musculus*, *Arabidopsis thaliana*, *D. melanogaster*, and *C. elegans*. Empirical evaluation revealed that, on human data, COME attained AU-ROC score of 99% which outperformed other predictor performance by 1%. Furthermore, it achieved the accuracy of 95%, 95%, 99%, 98%, and 99% on Mouse, Worm, Fly, and Plant species test sets, which overall proved better than other computational predictors. Despite the emergence of several tools, the task to identify various classes of RNAs among a collection of fully reconstructed transcripts was considered a tough task. In this regard, Valentin et al. [64] proposed an alignment free tool called flexible extraction of lncRNA (FEELnc) that correctly annotated lncRNA using a random forest classifier trained with generalized features including open reading frame and k-mer frequencies. Performance comparison with five state-of-the-art tools proved that FEELnc either managed to surpass or at least marked similar classification performance over datasets extracted from NONCODE [41] and GENCODE [68] databases. FEELnc also facilitated a special fine-tuning module through which users formalized lncRNAs annotations and identified lncRNAs despite the absence of training instances of lncRNAs.

Several researchers have reaped the advantages of multiple models through ensemble learning to achieve better performance in diverse bioinformatics tasks [71,72,73,74]. Building on the wide success, a number of ensemble learning approaches have been developed to identify coding and non-coding RNAs.

Predominantly, ncRNA identification approaches utilize only sequence derived features that hinder the achievement of stable performance across different species due to a large fluctuation of sequence characteristics. Han et al. [75] combined sequence intrinsic features, physicochemical property based features, and secondary structure features to develop an “LncFinder” predictive framework. LncFinder [75] developed a meta-classifier using five different classifiers including Logistic Regression, Support Vector Machine, Random Forest, Extreme Learning Machine, and Traditional Neural Network. Authors compared the performance of LncFinder with CPC [57], CPAT [60], CNCI [55], PLEK [56], and CPC2 [58] over human (Homo sapiens), wheat (Triticum aestivum), mouse (Mus musculus), chicken (Gallus gallus), and zebrafish (Danio rerio) datasets. Empirical evaluation indicated that LncFinder achieved top accuracy of 97, 94, 93, and 88 on human, chicken, mouse, and zebrafish datasets, outperforming previous best performance by 1%, 2%, 1%, and 2% on four different species datasets.

Xu et al. [76] proposed an iterative ensemble learning paradigm namely “LncPred-IEL” based on transcript and sequence derived features. Authors segregated open reading frame length, coverage, integrity, Fickett score, hexamer score, gravy, instability, composition–transition–distribution, spectrum, mismatch, reverse compliment K-mer, pseudo nucleotide composition, and auto-cross variance features into six distinct groups. They also utilized a feature selection approach to optimize each group where they applied analysis of variance followed by minimal redundancy maximal relevance approach to discard redundant features and retain only the most discriminative features. They constructed independent base predictors (Random forest) using a specific set of features and utilized an iterative supervised paradigm to combine the best performing models. Authors compared the performance of LncPred-IEL [76] with existing computational predictors using datasets of four different species including Human, Mouse, Fruitfly, and Zebrafish. In addition, they evaluated LncPred-IEL [76] on two newly developed Human and Mouse species datasets. On four benchmark datasets, they compared the performance of LncPred-IEL [76] with four other predictors including CPAT [60], CPC2 [58], CPPred [59], and LongDist, proposed the LncPred-IEL [76] approach that achieved the accuracy of 90%, on the Human dataset and 92% on the Mouse dataset, outperforming the previous best performance by 2%, whereas, training the model on a full newly developed human dataset, LncPred-IEL [76] marked the performance of 90% and 85% when tested on the fruitfly and zebrafish testing datasets, respectively. Similarly, training the model on a full newly developed Mouse dataset, LncPred-IEL [76] achieved the performance of 96% for fruitfly and 91% for zebrafish, respectively.

Liu et al. [77] developed a stacked ensemble-learning methodology “PredLnc-GFStack” to discriminate ncRNAs from protein-coding transcripts. Unlike existing approaches, PredLnc-GFStack [77] utilized a novel feature selection algorithm where sequence derived features of six different categories were passed to a genetic algorithm which extracted optimal features using an area under receiver operating characteristic score produced by a random forest classifier. Optimal features were passed to a multiple random forest model which operated on a different subset of features. For final prediction, multiple random forest models were stacked on top of each other to identify ncRNAs. Authors evaluated the performance of PredLnc-GFStack [77] on two newly developed Human and Mouse species datasets where PredLnc-GFStack achieved accuracy of 90% and 91%. They also performed cross-species evaluation using benchmark test sets. Training fully on a newly developed Human dataset and testing on five species datasets, PredLnc-GFStack [77] marked the accuracy of 97% on Human, 94% on Mouse, 90% Zebrafish, 94% on Fruitfly, and 96% on *S. cerevisiae* datasets. Likewise, training fully on Mouse datasets and testing on five species test sets, PredLnc-GFStack [77] achieved the accuracy of 88%, 94%, 84%, 92%, and 94%, respectively.

In order to increase the poor performance of RNNs while dealing with small sized open reading frames (sORF), Chen et al. [78] developed coding potential estimation “CPE-SLDI” methodology. Taking the deficiency of coding RNA sequences having small sized open reading frames into account, authors utilized an over-sampling technique to augment protein-coding transcripts and integrated diverse features including open reading frame length, coverage, integrity, Fickett score, hexamer score, gravy, instability, and composition–transition–distribution to feed an extreme gradient boosting meta-classifier. They compared the performance of CPE-SLDI [78] with multiple baseline data augmentation approaches and machine learning classifiers. To prove the integrity of proposed CPE-SLDI [78], they compared the performance with six existing computational predictors (CNCI [55], CPC2 [58], PLEK [56], CPPred [59], and CPAT [60]) using four datasets Human, Mouse, Human-sORF, and Mouse-sORF. CPE-SLDI achieved the accuracy of 97%, 84%, 97%, and 75% on Human, Human-sORF, Mouse, and Mouse-sORF datasets, outperforming previous best performance by the figure of 3%, 3%, 1%, and 1%, respectively.

Considering the wide success of deep learning in extracting long range hidden relationships of residues [79], multiple deep learning approaches based on Convolutional Neural Networks (CNNs), Deep Stacking Networks (DSNs), Recurrent Neural Networks (RNNs), and Deep Belief Network (DBNs) have been proposed to distinguish ncRNA from protein coding transcripts.

Fan et al. [80] developed “LncRNA-MFDL” methodology which integrated multiple features including open reading frame, secondary structure, the most like coding domain transcript, and K-mer to feed a deep neural network for the accurate identification of ncRNAs. Empirical evaluation on Human genome dataset revealed that LncRNA-MFDL [80] attained the performance of 97%, which outperformed CPC [57] by 6%, CNCI [55] by 4%, and the LncRScan-SVM [50] approach by 3%. In cross-species evaluation, LncRNA-MFDL [80] achieved the performance of 96%, 91%, 96%, 93%, 96%, 87%, 89%, 90%, 97%, and 90% for testing datasets of Anole lizard, Zebrafish, Chicken, Gorilla, Macaque, Mouse, Lamprey, Orangutan, *Xenopus*, and *C. elegans*. Tripathi et al. [81] developed the ncRNA identification tool “DeepLnc” which utilized K-mer frequencies of sequence residues as features and a bag of tricks based deep neural network as a classifier. Using RefSeq [67] and LNCipedia [82], authors developed ncRNA and coding RNA datasets. Empirical evaluation on the benchmark dataset indicated that DeepLnc effectively handled nonlinearity in data with the use of fewer parameters and attained accuracy of 99% on Human genome datasets for the task of distinguishing ncRNAs from protein-coding RNAs.

Considering the non-availability of computational tool capable to identify ncRNA along with their functions, Yang et al. [83] presented “LncADeep” methodology based on deep neural network and deep belief network, which was capable of discriminating ncRNAs from coding RNAs as well as annotating biological functionality. LncADeep [83] acquired intrinsic features from sequences including open reading frame length, hexamer score, and Fickett score to feed the deep belief network. For the task of functional annotation, the first ncRNAs interaction with proteins was estimated by feeding sequence and structural information to the deep neural network. To effectively handle full length and partial length transcripts, authors developed three separate models using full and partial length transcripts, only partial length transcripts, and solely full length transcripts. To prove the effectiveness of LncADeep [83], they compared the performance of LncADeep with four existing predictors COME [63], lncScore [61], lncRScan-SVM [50], CPC [57], CNCI [55], CPAT [60], CPC2 [58] FEELnc [64], PLEK [56], longDist [49], and lncRNA-MFDL [80]. Empirical evaluation revealed that, for ncRNA identification, over both Human and Mouse datasets, it achieved the top specificity of 97% and 96% using full-length transcripts, outperforming other predictors’ performance by 1% and 4%.

Baek et al. [84] proposed a hybrid predictive methodology “LncRNAnet” to discriminate ncRNAs from coding RNAs. LncRNAnet [84] used one-hot encoding to learn statistical representation of sequences, RNN to extract dependencies of residues, and CNN to deeply explore different stop codons for the extraction of open reading frame indicators. Authors compared the performance of LncRNAnet with four existing computational predictors including CPAT [60], CNCI [55], CPC [57], and PLEK [56] using Human, Mouse, and 11 cross-species datasets such as Chicken Frog, Fruitfly, Zebrafish, Chimpanzee, Cow, Gorilla, Orangutan, Pig, Platpus, and Rhesus. Empirical evaluation indicated that LncRNAnet achieved the accuracy of 0.92% on Human and Mouse datasets to outperform previous best performance by 6%, whereas it achieved the accuracy of 0.9300%, 0.8965%, 0.9085%, 0.8980%, 0.9165%, 0.9320%, 0.9085%, 0.9335%, 0.9335%, 0.9050%, and 0.9270%, and outperformed other predictors’ accuracies by an average amount of 3%. Extrinsic evaluation revealed that LncRNAnet achieves better performance for short length sequences primarily due to a one-hot encoding scheme. Because a one-hot encoding scheme lacks capturing order, positional information of residues as well as facing the issue of the curse of dimensionality on the account of long sequences that eventually derail the generalizeability of the classifier. Furthermore, Hill et al. [48] developed a gated recurrent neural network based methodology, namely “mRNN”, to discriminate ncRNAs from coding RNAs. Using a bag of neural tricks (e.g., dropout), mRNN [48] managed to interpret long-range dependencies and contextual information of residues which assisted gated recurrent units to accurately identify ncRNAs solely using one-hot encoded sequence features. Using Human, Human–Challenge, and Mouse species datasets taken from GENCODE [68], empirical evaluation, and comparison of mRNN [48] with CPAT [60], FEELnc [64], and longdist-SVM [49] indicated that mRNN achieved performance of 98%, 96%, and 95%, which outperformed existing predictor performance by 4%, 16%, and 2% on respective datasets in terms of accuracy.

Dang et al. [85] utilized the differences present in the distribution of k-mer frequency to generate the k-mer occurrence matrix. Optimal combination of k-mer and convolutional neural network model performance is compared with four machine learning classifiers including RF, SVM, LR, and DT using Human, Mice, and Chicken datasets. Empirical evaluation revealed that proposed deep learning methodology achieved the top accuracy of 0.99%, 100%, and 0.88% over Human, Chicken, and Mice datasets, outperforming machine learning classifiers by 11%, 7%, and 7% on respective datasets.

In a nutshell, discrimination of long non-coding RNA from protein-coding RNAs is primarily based on three aspects: first is the segregation based on the length of the open-reading frame of coding and non-coding RNA sequences, second is the categorization by estimating how similar sequences are to known protein sequences, and third is the inference through the conservation of secondary structure information. A precise categorization of such methodologies in terms of features, alignment approaches, classification model, target species, peak performance values, and availability of source code is provided in Table 1 and Table 2.

### 2.2. Identification of Long Intergenic RNAs

Long ncRNA is further categorized into various sub types where each sub type has distinct biological roles [86]. Long intergenic (lincRNAs) is one of the sub types of long ncRNA and has been discovered in the genomes of the mammals through analyzing transcriptomic data. LincRNAs mimic the length of lcRNA (200 bp) and are considered noteworthy resources in gene transcription, as well as translation [87]. Studies have proved that almost 93% of complex diseases relevant to single nucleotide polymorphisms have strong connections with intergenic regions [88]. Furthermore, lincRNAs are also responsible for multiple myeloma [89,90], and multifarious cancers [90]. Although researchers have managed to discover a substantial number of lincRNAs; however, their core functionalities are still yet to be decoded. LincRNAs have a structure similar to the coding RNAs of exons and introns; nevertheless, lincRNAs neither have a long ORF nor perform protein coding.

According to our best knowledge, there exists only one computational approach for the identification of lincRNAs. Yu et al. [87] developed a deep learning methodology for the identification of lincRNAs. Proposed methodology utilized a multi-layer autoencoder to learn optimal representation of sequences. Optimized representation of sequences was later fed to a predictive neural network for the identification of lincRNAs. In order to evaluate the integrity of the proposed approach, they compared the performance of an auto-encoder based model with the most widely used machine learning classifier support vector machine (SVM) and standard neural network model. Empirical evaluation on a newly developed dataset indicated that the auto-encoder based deep learning model outperformed the SVM and trivial neural network based models, achieving almost 100% performance on a newly developed Human lincRNA dataset.

### 2.3. Distinguishing Circular RNAs from Long Non-Coding RNAs

Another novel sub type of long ncRNA is Circular RNA (circRNA), which is generated by the process of back-splicing. CircRNAs can be broadly classified into an intronic containing a single intron and an exonic containing a flanking intron and exon (ccRNA). CircRNAs exist in almost 10% of genes due to back splicing, which acts as a modulator in microRNA activity. It has been established that circRNAs are abundantly expressed in plasma and tumor tissues of breast cancer patients where they regulate gene expression impacting metastasis, chemoresistance, and proliferation of breast cancer through specifically regulating and binding microRNAs expression [91]. Gene regulation capability of circRNAs linked them up with human diseases like lung cancer [92,93]. Considering the potential of circRNAs acting as prognostic markers, diagnostic markers, and therapeutic targets for diverse diseases [91,92,93], researchers have extensively explored the characteristics, functions, and regulatory paradigms of circRNAs by distinguishing them from other lncRNAs. According to our best knowledge, to date, there exist six approaches [94,95,96,97,98,99] that are capable of discriminating circRNs from other lncRNAs. A broad overview of these approaches in terms of features, representation scheme, classification algorithm, target species, and peak performance is given in Table 3.

The first approach PredcircRNA proposed by Pan et al. [94] generated statistical representation of raw RNA sequences by combining seven different features including graph features, sequence composition, conservation information, tandem repeat, ALU, ORF features, and SNP density. Based on this statistical representation, the classifier managed to acquire a linear weight combination of multiple kernels in which every kernel converting the hands-on representation into a higher dimensional space just to make the data linearly separable. Afterwards, SVM was utilized for final classification, the performance of which was compared with two other classifiers: Random Forest and Multi-Kernel Learning classifier. Among all of these, SVM based predictive methodology PredcircRNA attained a top accuracy of 86% on the benchmark dataset. Using a similar set of features, Chen et al. [95] developed H-ELM, which additionally utilized minimum redundancy maximum relevance (mRMR) as well as the iterative features selection approach to perform a deeper analysis of sequences and retain only the most discriminative set of features. Using most informative features and a hierarchical extreme learning classifier, authors distinguished circRNAs from other lncRNAs. A major bottleneck of both discussed approaches is their inability to acquire underlay structure and formation of circular RNA along with a lack of utilization of trinucleotide co-occurrence information. In addition, the dependence of these approaches over several manually curated features also make them less adaptive and ineffective.

Sun et al. [96] developed “CircCode” to precisely identify translated cicRNAs in Human as well as *Arabidopsis thaliana*. Empirical evaluation on two different datasets indicated that CircCode greatly minimized false positive rate. CircCode was exposed as a web service to facilitate diverse users. Niu et al. [97] developed a novel methodology “CirRNAPL” which fused nucleic acid composition features with circRNA sequence features to acquire rich inherent relationships of residues. CirRNAPL utilized an extreme learning machine classifier based on a particle-swarm optimization approach to accurately distinguish circRNAs from Human lncRNAs, protein-coding transcripts, and stem cells. Empirical evaluation on three benchmark datasets, a detailed performance comparison with baseline classifiers and existing predictors, indicated that CirRNAPL archived the top accuracies of 81%, 80%, and 78% on three core datasets, respectively.

Dominantly, computational predictive methodologies make use of manual curated features; however, such approaches extract redundant and irrelevant features as well. Building on these deficiencies, Chaabane et al. [98] developed a hybrid end-to-end computational framework “circDeep” to accurately discriminate circRNAs from lncRNAs. They developed three different descriptors including reverse complement matching to determine the predictive potential of circRNA sequences, for residue context, conservation descriptor to conserve species and motif specific information, and a neural embeddings based asymmetric convolutional neural network with Bidirectional Long Short-Term Memory network (ACNN-BLSTM) to extract local features and retain long range dependencies. Using two benchmark datasets, performance comparison with existing computational predictors H-ELM [95] and PredcircRNA [94] indicated that the proposed circDeep approach attained the top MCC of 85%, which outperformed existing approaches by a significant margin of 12%. Although authors managed to raise the classification performance significantly, the extraction of a reverse complement matching score is very time-consuming and also depends on two manually curated features.

To further increase the predictive performance by effectively handling redundant and irrelevant features, more recently, Stricker et al. [99] developed an end-to-end deep learning framework CircNet to automate the process of extracting important sequence features for the task of discriminating CircRNAs from other long ncRNAs. CircNet utilized encoder–decoder architecture based convolutional operations to obtain bottleneck representation of sequences. CircNet utilized another convolutional operations based architecture to acquire most discriminative features from bottleneck sequence representation to feed the final classification layer. Authors performed extensive experimentation with different regions of circRNA sequences to reveal which region contained the most significant residue distribution for circRNA identification. Preserving the important sequence information regarding CircRNA identification, CircNet attained a top accuracy of 98% on the benchmark dataset. In comparison to CircDeep [98], H-ELM [95], and PredcircRNA [94], CircNet outperformed the state-of-the-art CircRNA identification approach by a significant margin of 10% in terms of F1-score.

### 2.4. Identification of Small Non-Coding RNAs

Small ncRNAs possess a length of around 20–30 bp and are involved in translation, splicing, and regulation of genes [100]. Primarily, small ncRNAs are segregated into sub-classes, where every subclass has distinct biological significance [101,102].

One of the unique classes of small ncRNA molecules is piRNA molecules, which not only exist in germline cells of animals but also in diverse human somatic cells [103]. Their sequence length falls in the range of 26 to 32 nucleotides [104,105,106]. Studies have revealed that piRNA plays an important role in a variety of gene functions including protein translation, preserving genome integrity, transposon silencing, and gene expression regulation [107,108]. PiRNA molecules move inside the genome, and also induce insertions, deletions, and mutations that might produce genome instability [109]. Similarly, studies [110,111,112,113] have reported that piRNA occurrences are also strongly linked with a variety of tumor varieties, where they contribute to the development and acceleration of cancer cells.

Considering these promising findings, there is an immense interest to discover and categorize piRNA molecules along with the study of functions related to gene stability, drug development, diagnosis, and treatment for cancer cells. In order to categorize RNA sequences into pi and non-pi RNA sequences, several methodologies came into the picture. A broad overview of these approaches in terms of features, representation scheme, classification algorithm, target species, and peak performance is given in Table 4. For example, in 2007, Betel et al. [114] proposed the very first one-layer machine learning methodology for the identification of piRNA molecules in Mouse species. Their proposed methodology utilized position specific residues properties to generate sequence representation and support vector machine (SVM) for classification. Empirical evaluation revealed that, across the Mouse species dataset, their proposed machine learning approach manages to achieve a precision of 61%. In 2011, Zhang et al. [115] presented a piRNAPredictor that used the k-mer approach as feature representation and a machine learning classifier, namely support vector machine (SVM) for prediction. Evaluation of the proposed one-layer piRNAPredictor over five species datasets including rat, mouse, human, fruit fly, and nematode indicated the dominance of piRNAPredictor as compared to existing predictive methodologies by attaining a top precision of 90%.

In 2014, Wang et al. [116] utilized triple elements which combined structure and sequence information to learn rich representation of piRNA sequences and SVM classifier to develop a robust piRNA predictor, namely “Piano”. Performance analysis over four species human, mouse, rat, and Drosophila indicated that Piano outperformed existing piRNA predictive methodologies by attaining the top performance around 95% in terms of four most widely used evaluation metrics. In the same year, Brayet et al. [117] developed another one-layer predictive methodology “PiRPred” which used K-mer sequence descriptor and multiple kernels based SVM for the identification of piRNA molecules. Empirical evaluation over two species—Human and Drosophila—revealed that PiRPred raised the previous best performance by a decent margin by attaining the accuracy of 89%, sensitivity of 83%, and specificity of 95%. Liu et al. [118] developed “Pibomd” methodology for piRNA classification using sequence motifs as features and SVM as a classifier. Authors evaluated the integrity of Pibomd over five different species including rat, mouse, human, fruit fly, and nematode, where Pibomd managed to achieve the top performance of 91%, 92%, and 90% in terms of accuracy, sensitivity, and specificity.

In 2015, Menor et al. [119] developed “McRUM” methodology which utilized correlation based feature selection to extract important K-mer features and L1 based Gaussian kernel oriented SVM for final prediction. McRUM was evaluated over *Caenorhabditis elegans*, *Locusta migratoria*, and *Drosophila melanogaster*, where it achieves the top accuracy of 93%. In 2016, Lie at al. [120] proposed a weighted K-mer and SVM based approach for piRNA classification. Authors evaluated the performance of proposed machine learning piRNA predictor using datasets of four different species including human, mouse, drosophila, and rat where proposed predictors achieved the sensitivity and precision of 90%.

In 2016, Luo et al. [128] presented an ensemble learning methodology which utilized physico-chemical properties based encoding scheme and Random forest classifier to predict piRNA molecules. Performance assessment on three different species Human, Drosophila, and Mouse indicated that proposed predictive methodology achieved the performance of 96% across most widely used evaluation metrics. In the same year, Li et al. [127] presented “GA-WE” methodology which used multiple K-mer related features and weighted random forest classifier for piRNA classification. Authors evaluated the integrity of GA-WE on three different species Human, Drosophila, and mouse, where GA-WE outperformed the previous best performance by a decent margin as it achieved a top accuracy of 96%.

In 2017, Chen et al. [122] developed “piRNAdetect” methodology which used n-gram model based features and an SVM classifier for accurate identification of piRNA molecules. Authors evaluated the performance of piRNAdetect over *H. sapiens*, *R. norvegicus*, and *M. musculus* species where piRNAdetect managed to achieve accuracy of 84%. Around a similar time, Boucheham et al. [124] presented “IpiRId” methodology which utilized K-mer specific motifs as features to feed an SVM classifier based on the fusion of multiple kernels for accurate identification of piRNA molecules. IpiRId was evaluated over three species datasets including Human, Mouse, and Fly, where IpiRId attained a top accuracy of 94%. Pian et al. [129] developed a deep learning based piRNA classification approach namely “V-ELMpiRNAPred” which combined short sequence motifs with K-mer features to feed voting based extreme learning machine (V-ELM) classifier. V-ELMpiRNAPred correctly identified human piRNAs with an accuracy of 95%.

In 2018, Wang et al. [130] developed the first advanced neural architecture based methodology “piRNN” for piRNA classification. piRNN utilized position specific K-mer features to feed a convolutional neural network. Performance evaluation over four different species including *Caenorhabditis elegans*, *Drosophila melanogaster*, rat, and human revealed the dominance of piRNN which outperformed existing piRNA predictors by obtaining an accuracy of 95%, sensitivity and specificity of 97%. Around a similar time, Monga et al. [123] developed another one-layer approach “piRNApred” which utilized physico-chemical properties to capture biological characteristics of piRNA sequence residues and an SVM classifier for distinguishing piRNA molecules from non-piRNA molecules. piRNApred performance was analyzed over *H. sapiens*, *M. musculus*, *D. melanogaster*, *C. elegans*, *Danio rerio*, *Gallus gallus domesticus*, *Xenopus tropicalus*, and *Bombyx mori* species. Experimental results revealed that piRNApred attained a top accuracy of 99% which outperformed previous best performance by a significant margin.

All of these methodologies categorized RNA molecules into piRNA and non-piRNA classes. However, they neglected to discover their functions in the context of mRNA deadenylation. To address this problem, Lu et al. [121] presented a two-layer machine learning based predictor, namely “2L-piRNA”, in which pseudo K-tuple nucleotide composition was combined with physicochemical properties of nucleotides to learn rich inherent relationships of residues. Optimized sequence representation was passed to an SVM classifier which identified piRNAs at the first layer with an accuracy of 86% and predicted their functions at a second layer with an accuracy of 78% for the *M. musculus* species. Likewise, in the following year, Chen et al. [125] developed another “2L-piRNAPred” which combined a transcript composition based feature, position specificities of nucleotides, proportions of peptide sequence, and physicochemical properties to generate a comprehensive representation of piRNA sequences. 2L-piRNAPred utilized an SVM classifier to identify piRNA molecules at the first layer with an accuracy of 89% and piRNA function at the second layer with an accuracy of 84% over the *M. musculus* species dataset. However, both presented approaches “2L-piRNA” [121], and “2L-piRNAPred” [125] utilized SVM in the first layer, which failed to correctly distinguish piRNA and non-piRNA molecules along with function kinds mainly due to the fact that piRNA molecules and non-piRNA molecules were highly similar to each other. In addition, these methodologies required human expertise to effectively extract most relevant features, which is a major downfall [132].

Building on these deficiencies and considering the room for improvement, Khan et al. [131] presented another 2-fold computational predictor, namely “2L-piRNADNN”, for the identification of piRNA molecules and their function types. They combined di-nucleotide auto covariance features with six physico-chemical properties based features to generate rich sequence vectors. piRNA sequence vectors were passed to a deep neural network which automatically extracted most informative features for the task of piRNA classification and piRNA function prediction. Empirical evaluation of proposed methodology on the *M. musculus* species dataset indicated that 2L-piRNADNN achieved the accuracy of 92% at the first layer and 85% at the second layer.

Recently, Zuo et al. [126] developed a robust two-layered integrated classification methodology, namely “2lpiRNApred”, which identified piRNAs in the first layer and estimate their function for the induction of target mRNA deadenylation in the second layer. They investigated five sequence descriptors including Kmer, Geary auto-correlation, Normalized Moreau Broto auto-correlation, General parallel correlation pseudo dinucleotide composition (PDC), and general series correlation PDC. To discard redundant features, they developed a novel feature selection algorithm using Luca fuzzy entropy and a Gaussian membership approach. They investigate the performance of Sparse Representation and an SVM classifier to construct 2lpiRNApred methodology for accurate classification of piRNA molecules. Empirical evaluation on the *M. musculus* species dataset revealed that 2lpiRNApred attained the accuracy of 88% at the first layer and 81% at the second layer.

Other than PiRNA, some of the initially found subclasses of small ncRNAs are transfer RNAs (tRNAs) and ribosomal RNAs (rRNAs). Contrary to these primary subclasses, small ncRNA has a few other subclasses such as Group 1 introns, and 5S rRNA which were included in the list lately. SnoRNA, and miRNA usually play their part in cancer by a sequence of mechanisms [133]. SnoRNAs escort chemical modifications in transfer RNAs (tRNAs), ribosomal RNAs (rRNAs), and other small nuclear RNAs. Primarily, snoRNA has two core classes: HACA-BOX and CD-Box. Meanwhile, HACA-Box has strong connections with methylation, and CD-Box is linked with pseudouridylation. However, the impact of pseudouridylation and methylation modifications on the working of mature RNAs is yet to be discovered. These modifications are usually known to magnify RNA folding and exchange with ribosomal proteins. Unlike snoRNAs, scaRNAs are body specific RNAs that are localized to nuclear organelles and Cajal bodies. Most ScaRNAs are not only functionally but also structurally identical to snoRNAs; however, few of them are considered composites of HACA-Box and CD-Box, which can direct modifications in both pseudouridylation and methylation—whereas, miRNAs mainly perform post transcriptional gene expression regulation and RNA silencing. MiRNAs target almost 60% of human genes as they exist in abundance. MiRNAs play an indispensable role in several biological processes like cell differentiation, proliferation, and death [134,135,136,137]. Studies have proved that miRNAs are involved in diverse complex human diseases such as neurodegenerative, cancer, autoimmune, and cardiovascular diseases [26].

Ribosomal RNA (rRNA) is essential for all living organisms as it plays a key role in protein synthesis. rRNA characteristics are considered extremely valuable for the development of multifarious antibiotics. In addition, 5S ribosomal, another kind of rRNA, also exists in ribosomes. Although its function has not been discovered yet, it has been seen that their deletion substantially alleviates protein synthesis and also produces detrimental effects on the fitness of the cell [138]. Likewise, 5.8S ribosomal RNA actively participates in protein translocation [139]. It also forms covalent connection with tumor suppressor proteins [140] and can be used to detect miRNA [141], and understand other rRNA pathways and processes in cells [142].

Two sub-classes of nc-RNA (transfer RNA (tRNA) and ribosomal RNA (rRNA)) play an important role during translation events in which proteins are formed [143]. Another well known class of nc-RNA is microRNAs (miRNAs) which participate in regulation of various biological processes such as proliferation, differentiation, stress tolerance, apoptosis, energy metabolism, immune response, and cell cycle [144]. It consists of 19–25 nucleotide long regulative RNA molecules [144,145,146]. microRNAs always look for opportunities to bind to other RNAs that resemble them. In such a way, they stop activities of binded RNAs, which leads to preventing the formation of proteins most of the time [147]. In cancer, there are some well established famous genes known as oncogenes, that can turn on in cancer cells and promote cell division that gets out of control. microRNAs have the ability to control and shut off the process of oncogenes [147]. Recent research has proven that, in a cell where microRNA is present, oncogenes would be shut off and cells will not divide. On the contrary, if microRNA genes are missing from a cell, oncogenes could be used for the promotion of cancer [148].

Park et al. proposed a deep learning based approach called Deep RNN [149]. Deep RNN makes use of a recurrent neural network based approach for the identification of microRNAs. Deep RNN requires sequence alignment and extraction of a secondary structure of the sequence at the pre-processing stage. Secondary structure based features are passed to RNN layers that extract more discriminative features (Table 5).

### 2.5. Segregating Small and Long Non-Coding RNAs

With the advancements of biological research, it was extrapolated lately that most of the genome of living organisms are transcribed into multifarious ncRNAs, and they perform multifarious essential biological functions [154,155,156]. After these findings, detecting new ncRNAs and discovering their biological functions became a promising area of research [39,157,158]. Furthermore, to understand the behavior and role of ncRNAs in biological operations, various sub-classes of ncRNA have been identified [159]. Based on folding of nucleotide sequence, sequence length, and their biological role, ncRNAs are categorized into small and long ncRNAs.

Lertampaiporn et al. [160] proposed an ensemble approach for the classification of ncRNAs into two sub-classes called long ncRNA and small ncRNA. Through the proposed ensembling approach, they reaped the benefits of two classifiers: random forest and logistic regression. They used a set of five features (sequence, modularity, coding potential, structure, and structural robustness) to represent a sequence. They used a correlation based feature selection algorithm to discard less discriminative features from the set of extracted features.

### 2.6. Family Classification of Small Non-Coding RNAs

In order to accurately classify small non-coding RNAs into respective families, a number of computational methodologies have been developed. In Table 6, we have summarized state-of-the-art machine and deep learning based methodologies proposed for the classification of small non-coding RNAs.

Antonino Fiannaca [159] developed a deep learning based methodology where secondary structural features of RNA sequence and convolutional layers were used for the extraction of more discriminative features to feed dense layers for classification of small ncRNA. Their methodology achieved 81% accuracy for classification of small ncRNA sequence into 13 predefined classes. Another interesting methodology was proposed by Emanuele Rossi, which used graph convolutions for the extraction of discriminative features from secondary structural features of small ncRNA sequences. Features extracted by graph based convolutional layers were passed to fully connected layers for the classification of small ncRNA sequences to 13 different classes with 85% accuracy [161].

Instead of considering a secondary structure as a key determinant to determine small ncRNA function, Noviello et al. [162] presented a deep learning methodology based on just raw sequence information. To extract discriminative high level features from small ncRNA sequences represented using k-mer binary encoding, they used a three-layer convolutional neural network (CNN) and showed that raw sequence information is enough to determine the function of small ncRNA. In order to optimize proposed CNN model, they performed experimentation with different kinds of padding schemes, K-mer encodings (e.g., 1-mer, 2-mer, 3-mer), CNN layers, and bag of tricks (e.g., Dropout). They found that K-mer encodings are not very much affected by padding criteria (e.g., random, constant, new symbol padding) and constant or new-symbol padding is more prone to impact overall predictive performance. K-mer encoding scheme handles the noise better as compared to a trivial one-hot encoding scheme. Furthermore, increasing the depth of CNN improves the predictive performance and dropout strategy largely assists the CNN model for distinguishing functional and non-functional ncRNA sequences. Building on these findings, the optimized CNN model outperformed existing secondary structure based approaches in terms of discriminating function and non-functional small ncRNA sequences and classification speed, indicating the suitability of purely sequence information based predictive methodologies for large scale genome annotation. A performance comparison with baseline RNN and state-of-the-art predictive methodologies showed that the proposed CNN model achieved the top accuracy of 96% on the benchmark dataset, outperforming previous best performance by a significant figure of 10%.

Likewise, considering the room for improvement in secondary structure based predictive methodologies for small ncRNA family classification, Chantsalnyam et al. [165] presented a deep learning methodology “ncRDeep” which extracted discriminative features using a simple yet efficient convolutional neural network model from one-hot encoded small ncRNA sequences. They optimized the training and generalizeability of ncRDeep using a bag of neural tricks such as batch normalization and dropout. Using only sequence information, ncRDeep achieved an accuracy of 88% on the benchmark dataset which outperformed previous best performance by 9%. Considering small ncRNA family classification approaches based on secondary structure related features usually just take global characteristics into account while neglecting mutual influence of local structures, Asim et al. [166] developed a robust and precise CNN based classification methodology “RPC-snRC” using only sequence information. They applied a maximum-length copy padding trick to generate fixed length ncRNA sequences where representation at character and higher order residue level is learned using a variety of encoding schemes. They utilized one-hot encoding, randomly initialized embeddings, and pre-trained residue embeddings to investigate whether deep learning approaches perform better with character level encodings or higher order residue encoding. Two recent based classification methodologies were treated as baseline. Using local and global residue property based sequence vectors, precise CNN effectively captured the essence of a small ncRNA sequence for the task of small ncRNA family prediction. Performance comparison of proposed RPC-snRC methodology with baseline and state-of-the-art computational predictors over the benchmark dataset showed that RPC-snRC methodology achieved an accuracy of 95%, which outperformed previous best performance by a great margin of 10%.

### 2.7. Computational Methodologies for Clustering of Non-Coding RNA

Classification methodologies can be categorized into two different types: supervised and unsupervised. In the supervised type, we provide class labels information along with sequences to train any machine or deep learning model. On the other hand, in unsupervised types, we do not provide class labels to train the model; it finds similarities among the sequences and makes clusters of similar sequences. Rather than classification of ncRNAs to predefined classes, various clustering based approaches have also been utilized. Clustering based approaches identified several new classes of ncRNAs. In Table 5, we have summarized state-of-the-art machine and deep learning based methodologies proposed for the clustering of non-coding RNAs. Saito et al. proposed an Unsupervised Learning approach (EnsembleClust) for ncRNA classification [150]. This technique required the input as unlabeled samples to construct ncRNA clusters. In this technique, ncRNAs were clustered on the basis of structural alignment scores. Authors performed sequence alignment using the Waterman algorithm [167], and a secondary structure was acquired using the McCaskill algorithm [168]. Another similar methodology was proposed by Tsuchiya et al. [152]. In the proposed approach, the authors utilized a read mapping profile alignment program that used decomposition for aligning and folding RNA sequences simultaneously (DAFS). This technique was more useful to discriminate ncRNAs located in the brain.

Another similar ncRNA detection approach known as RNAscClust was proposed by Miladi et al. [151]. This technique made the clusters of RNA sequences by using graph-based patterns and structure conservation. Authors provided two benchmark datasets: Rfam-ome and Rfam-cliques. The quality of predicted clusters was measured by rating how well it agrees with the sequences annotated in the true Rfam database.

### 2.8. ncRNA Classification Datasets

To assess to what extent machine and deep learning approaches are capable of discriminating different ncRNAs from protein-coding transcripts (PCTs) or each other, a number of datasets have been developed using public metathesauruses like RNA Central [169], ENCODE, RefSeq [67], ENSEMBL, and NONCODE [41].

**Figure 2:**
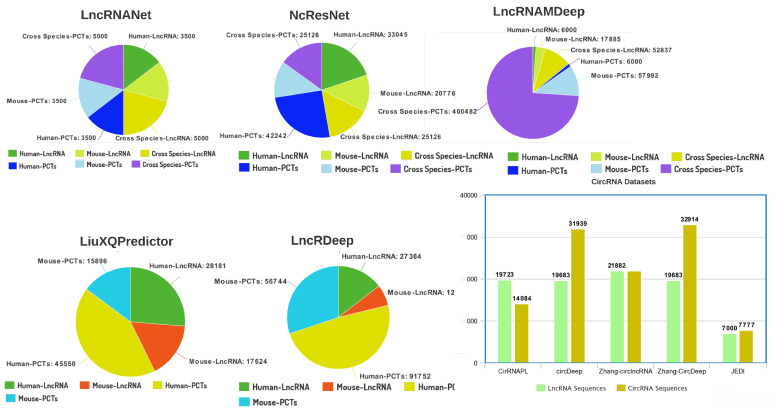
Statistics of different benchmark datasets used in the literature to evaluate the performance of ncRNA classification predictors.

A number of predictive methodologies including LncRNANet [84], NcResNet [170], LNcRNAMDeep [171], LiuXQpPredictor [172], and LncRDeep [83] utilized five different lncRNA datasets to distinguish lncRNA sequences from PCTs which contain Human, Mouse, and cross-species sequences. Sequence-to-genre distribution of five different lncRNA identification datasets is summarized in pie charts (Figure 2). Likewise, for CircRNA identification, CircRNAPL [97], circDeep [98], ZhangCircLncRNA [173], ZhangCircDeep [173], and JEDI [174] are evaluated on five distinct datasets containing lncRNA and circRNA sequences for the task of differentiating circRNA sequences from lncRNA sequences.

**Figure 3:**
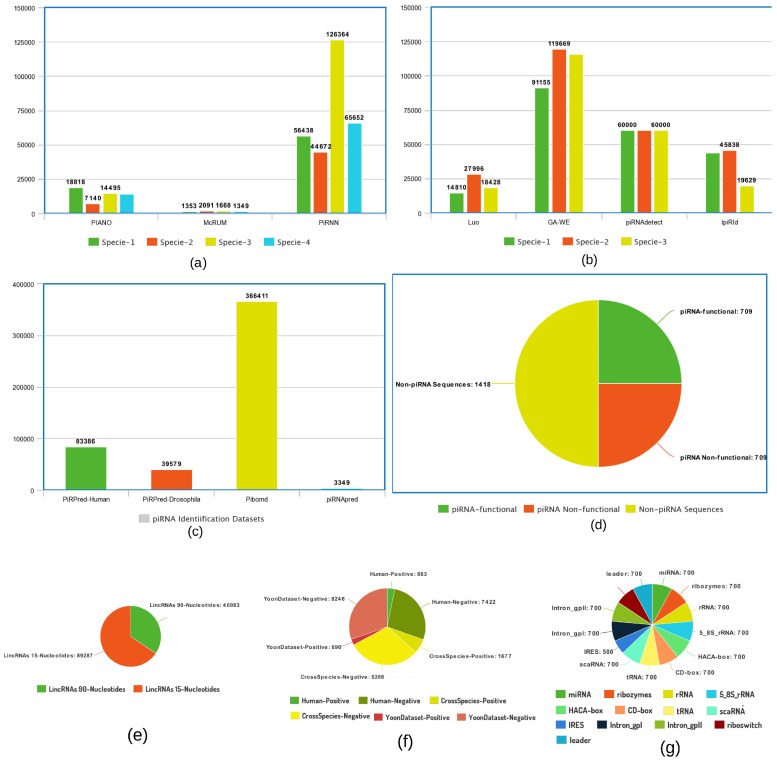
Statistics of different benchmark datasets used to identify piRNA (**a**) 1-Layer 4 Species Datasets, (**b**) 1-Layer 3 Species Datasets, (**c**) 1-Layer 2 and 1 Species Datasets, (**d**) 2-Layer Datasets, where Species = *H. sapiens*, *M. musculus*, *D. melanogaster*, *C. elegans*, *Danio rerio*, *Gallus gallus domesticus*, *Xenopus tropicalus*, *Bombyx mori* Table 4, LincRNAs (**e**), MicroRNAs (**f**), and family of small ncRNAs (**g**).

For small ncRNA, especially piRNA identification, there exist a variety of datasets belonging to distinct species. Figure 3a–d describes the aggregated statistics of different species datasets (whose names are mentioned in Table 4) along with respective computational predictors. For LincRNA identification, there exists only one benchmark dataset prepared by Yu et al. [87], the statistics of which are summarized in Figure 3e. Similarly, only the benchmark dataset used in literature for small non-coding RNA family classification is summarized in terms of 13 different classes, and the distribution of each class is given in Figure 3g. For microRNA identification, a positive and negative number of sequences with respect to three different benchmark datasets [149] are given in Figure 3f.

## 3. Sub-Cellular Localization of Coding and Non-Coding RNAs

ncRNAs contribute to several biological functions such as dosage compensation, genomic imprinting, and cell differentiation [26,27]. In addition, ncRNAs are strongly linked to several complex diseases including cardiovascular disorders, Alzheimer’s, and Cancer [26,27]. Over the period, researchers have discovered that localizations of ncRNAs within cells primarily determine their biological functions [175,176]. The identification and deep investigation of localization of ncRNAs through controlled biological experiments are extremely labor-intensive tasks and also infallible to errors. Building on this, developing diverse machine and deep learning based methodologies to automate the process of identifying ncRNA subcellular locations and discovering their biological functions soon became the hottest research area in Bioinformatics. Throughout this period, the primary focus of researchers has been to develop robust computational methodologies that can accelerate ncRNA structural and functional research, enabling the practitioners to have a better picture of various biomedical implications.

Utilizing the RNALocate database [45] and other resources such as ENCODE project [177] and the Ensembl database [46], up to now, researchers have proposed six computational methodologies for long ncRNA (lncRNA), four for messenger RNA (mRNA), and two for microRNA (miRNA) for the task of sub-cellular localization. The importance of lncRNA, mRNA, and miRNA and computational methodologies proposed to determine their biological functionalities through sub-cellular localization are briefly discussed below.

### 3.1. Messenger RNA Sub-Cellular Localization

Sub-cellular localization of messenger RNA (mRNA) plays a pivotal role in post-transcriptional regulation of genes. Messenger RNA localization mechanisms and their dependency on transcript structure have esoteric biomedical implications, hence their localization patterns are essential to explore in order to acquire the fundamental understanding of molecular biology. Although recent sequencing based robust technologies enable the identification of mRNA localities in the context of certain sub-cellular compartments, the mechanisms associated with specific sequence structures have been poorly understood. Building on this, Yan et al. [178] presented RNATracker, which utilized a neural network to predict the distributions of messenger RNA trasncripts in the context of a pre-declared collection of sub-cellular compartments. CNN is more suitable for performing automated parallel feature engineering through learning and Long Short Term Memory (LSTM) for discovering the correlations among different positions, capturing cooperative binding and sequence context by analyzing the sequential data. Considering these advantages of diverse neural networks, RNATracker integrated a number of state-of-the-art deep learning approaches including CNN, LSTM, and Attention mechanism to effectively leverage secondary structure and sequence information. RNATracker substantially outperformed baseline predictors and paved the way for the generation of testable hypotheses related to cis-regulatory and trans-regulatory molecules, and also to estimate the mutation impact on gene regulation.

Zhang et al. [179] developed a machine learning based methodology iLoc-mRNA for accurate determination of mRNA sub-cellular localization. They utilized binomial distribution to obtain a unique representation of mRNA sequences and variance analysis to select an optimal set of features. Using the support vector machine, the proposed iLoc-mRNA methodology managed to achieve a top accuracy of 90.12% on the benchmark dataset for sub-cellular localization of *Homo sapiens*. Garg et al. [180] developed another machine learning based methodology mRNALoc to infer the sub-cellular localization of mRNAs. mRNALoc utilized a pseudo K-tuple nucleotide composition descriptor to generate the encoding of mRNA sequences and support vector machine for classification. In comparison to existing mRNA sub-cellular localization predictors, using 5-fold cross validation, mRNALoc achieved the accuracies of 99%, 75%, 74%, 67%, and 65% for mitochondria, endoplasmic reticulum, nucleus, cytoplasm, and extracellular region sub-cellular locations across the benchmark dataset. For an independent test set, mRNALoc attained the accuracies of 99%, 69%, 69%, 64%, and 58% for mitochondria, nucleus, endoplasmic reticulum, cytoplasm, and the extracellular region, respectively.

In the real world of transcriptomes, mRNAs are usually localized to multiple compartments as indicated by the RNA sub-cellular localization metathesaurus [45]. Considering the lack of computational methodology capable of predicting multiple compartments of mRNAs, Wang et al. [181] developed a deep learning methodology “DM3LOC” to predict multi-label sub-cellular localization of mRNA sequences. Typically, CNN is utilized to acquire discriminative features, BI-LSTM is utilized to take spatial distances and orientation of residues into account, and attention layer is employed to assign greater weights to important regions. Such attention mechanism is called single head attention as just one attention-to-weight vector is used. Although a single head attention paradigm facilitates some intelligence for the interpretation of model, it may negatively affect the predictive performance as well. For instance, for a protein sub-cellular localization task, a non-attention neural architecture produced better performance than a single head attention paradigm. Considering the downfalls of single head attention, Wang et al. [181] utilized CNN architecture based on multi head self-attention in order to attend to several sub-cellular components simultaneously and capture the important global features generated by the combination of multiple sequence elements. Performance analysis on a benchmark dataset and independent test set indicated that a multi head attention paradigm helped DM3LOC to achieve better parallelization and predictive performance, outperforming all existing predictive approaches and a close competitor RNATracker in terms of speed and overall accuracy.

### 3.2. MicroRNAs Sub-Cellular Localization

MicroRNAs (miRNA), which are also referred to as short ncRNAs, significantly participate in a number of cellular processes of animals and plants including development, digestion, proliferation, and differentiation in organisms accompanied with contributions in post-transcriptional gene regulation [182]. A number of studies have discovered that miRNAs usually target diverse compartments of cells [183,184]. Mature miRNAs generally exist in distinct cellular segments of cytoplasm, which involve mitochondria, endoplasmic reticulum, and RNA granules. More recent findings suggest that some miRNAs also contribute to regulating nucleus and epigenetic function. Moreover, most miRNAs possess multiple locations in the cells which reveal their abundant localization patterns [176]. Sub-cellular localization of miRNA not only facilitates the interactions among proteins and RNA but also determine the action mode of miRNA to target mRNAs. Sub-cellular localization of miRNAs is fundamentally needed to regulate diverse scientific processes that generally occur inside sub-cellular infrastructures or organelles—for example, mitochondrial metabolism performed by mito-miRNAs and synaptic plasticity conducted by endosomal miRNAs. As compared to other ncRNAs, a very limited amount of work exists related to miRNA sub-cellular localization due to the distinct sub-cellular localization characteristics of miRNAs, lack of ontologies, and scarcity of miRNA functional annotations.

Up until now, only four methodologies, namely miRGOFS [185], MIRLocator [176], miRnALoc [186], and MirLocPredictor [187], have been proposed for the prediction of miRNA sub-cellular localization. MIRLocator utilized an attention based sequence to sequence a neural network with pre-defined information of label order to discover sub-cellular localities of human miRNA. Yang et al. [185] developed a novel approach “miRGOFS” to estimate functional similarity of miRNA molecules. miRGOFS adopted a naive GO semantic-similarity measure which computed closeness between GO descendants as well as common ancestors to weight the features on the basis of their statistical significance. Authors represented miRNA sequences in terms of correlation scores computed using different approaches. To make final miRNA sub-cellular localization prediction, the SVM classifier with RBF kernel was used. Over the benchmark dataset, miRGOFS achieved the F1-score of 61.2%, indicating that miRGOFS attained significant coverage of *Homo sapiens*’ miRNA molecules.

MIRLocator identified high-level noteworthy features that are hard to capture from miRNA sequences. Considering the fact that most miRNA have multiple locations in cells and sub-cellular compartments are biologically correlated, rather than treating sub-cellular localities as independent target labels, inherent linkages among locations are incorporated into MIRLocator output. Authors transformed the multi-label problem into a sequence to sequence problem to better capture the hidden correlations of sub-cellular localities and to best utilize prediction information of recent locations. The authors reported that the MIRLocator managed to produce promising performance with little input information and outshined the models that were utilizing manually curated features or trivial recurrent neural network based approaches. Meher et al. [186] developed another miRNA sub-cellular localization predictor “miRnALoc” based on pseudo di-nucleotides compositions, thermodynamic, and physico-chemical properties. miRnALoc utilized an SVM classifier optimized thorough Grid Search to accurately identify sub-cellular compartments of miRNAs. Authors eliminated 80% overlapping sequences from core datasets using the CD-HIT tool. To prove the integrity of the proposed approach, authors compared the performance of miRnALoc with six baseline classifiers including artificial neural network, random forest, naive Bayes, boosting, bagging, and k-nearest neighbor as well as existing computational miRNA localization predictors using core and independent datasets. Empirical evaluation indicated that miRnALoc achieved an AU-ROC score of 63–71% on the core dataset, and 50% localization of the independent test set was accurately predicted.

MIRLocator [176] utilized a sequence-to-sequence model and pre-trained k-mer embeddings. The prime focus of pre-trained neural embeddings is to capture the semantic information of higher order residues while neglecting the position of higher order residues. Apart from semantics, the position of k-mers is another key component that defines the function of RNA molecules. Recently, Asim et al. [187] developed a novel sequence descriptor kmerPR2Vec that fused positional information of higher order residues with randomly initialized higher order embedding. Using kmerPR2Vec statistical representation, they developed a deep learning based end-to-end predictive methodology “MirLocPredictor” which utilized CNN for accurate determination of miRNA sub-cellular localization. A rich performance analysis using the Recurrent Neural Network as baseline and existing predictive methodologies over the benchmark dataset indicated that the MirLocPredictor attained top performance, outperforming previous best performance by 19% and 18% in terms of recall and precision.

### 3.3. Long Non-Coding RNA Sub-Cellular Localization

Amongst all ncRNAs, long ncRNAs (lncRNAs) are highly prevalent and have the most diverse functional classes. LncRNAs are massive RNA transcripts (200 nucleotides) that are estimated to surpass protein coding genes inside the human genome [188]. Nevertheless, lncRNAs are badly preserved at the sequence level; this is why their functional annotation is quite difficult. LncRNAs perform a number of indispensable molecular functions at diverse sub-cellular locations [189]. LncRNAs transcripts may reveal different cellular localities involving the nucleus, chromatin, exomoes, and cytoplasm [190,191]. In addition, lncRNA have noteworthy functions in development [192,193], and metabolism of cells like chromatin modifications [194,195], genome rearrangements [196,197], genetic markers [198,199], transcription [200], translation [201], and regulation of the cell cycle [202]. Having the knowledge of lncRNAs localization assists with comprehending their biological functionalities. Sub-cellular localization of lncRNAs depend on several facets such as structural and sequence motifs [203].

Guednas et al. [204] developed a deep learning based methodology called DeepLncRNA to predict the sub-cellular localization of lncRNA by directly analyzing transcript sequences of lncRNA. They processed 93 strand-particular RNA sequences of cytosolic and nuclear fractions acquired from diverse cell types to discover deferentially localized long ncRNAs. The DeepLncRNA approach developed marked significant performance and authors also reported that, primarily, sequence motifs are deriving lncRNA sub-cellular localization.

Su et al. [205] developed a bioinformatics framework called “iLoc-lncRNA” to estimate the sub-cellular localities of lncRNAs through embedding features of 8-tuple nucleotides into generic Pseudo K-tuple Nucleotide Composition (PseKNC). The developed framework utilized binomial distribution methodology. Extensive jackknife tests revealed that the developed framework managed to outshine a state-of-the-art lncRNA sub-cellular locality predictor by a promising figure.

Cao et al. [206] proposed a machine learning based lncLocator methodology for long ncRNA sub-cellular localization. The lncLocator utilized k-mer based features along with high-level abstraction features extracted using unsupervised deep learning models. At the classification stage, lncLocator takes both kinds of features as input for two different classifiers: SVM and random forest. Separately training both classifiers with two different features produced four trained models that are used for the prediction of long ncRNA sub-cellular locations in different compartments.

Considering that sequence descriptors introduce significant bias and irrelevant features as well as generating encoding, the use of feature selection approaches soon became a frontier in the development of robust lncRNA sub-cellular localization prediction approaches. In this regard, Zhang et al. [207] developed a machine learning methodology “KD-KLNMF" for accurate determination of lncRNA sub-cellular localization. They utilized a data augmentation approach to balance the imbalance dataset. Dinucleotide based spatial autocorrelation and k-mer descriptors were used to generate the representation of lncRNA sequences. In order to discard redundant features, a dimensionality reduction approach called nonnegative matrix factorization based on Kullback–Leibler divergence was used. An optimal set of features was passed to support a vector machine classifier that predicted different sub-cellular compartments of lncRNAs. Performance analysis on the benchmark dataset and independent test set indicates that KD-KLNMF attained the accuracies of 97% and 92% in terms of jack-knife evaluation.

Ahmed et al. [208] proposed a machine learning based predictive methodology Locate-R for accurate determination of lncRNA sub-cellular localization. They learned that representation of lncRNA sequences uses a very simple technique based on n-gaped l-mers. In order to select the most representative features of sequences, they utilized a Pearson correlation coefficient approach, the output of which was passed to the support vector machine classifier. Performance comparison with existing computational predictors lncLocator [206] and iLoclncRNA [205] indicated that Locate-R outperformed previous best performance by 2%.

Likewise, Fan et al. [209] developed a machine learning based methodology “lncLocPred” to accurately determine the sub-cellular localization of lncRNAs. They utilized three different sequence descriptors including K-mer, Pseudo Dinucleotide Composition (PseDNC), and Local Structure–Sequence Triplet Element to represent lncRNA sequences. In order to select the most representative sequence features only, they developed a feature selection approach using binomial distribution, variance threshold, and F-score. Highly discriminative features were passed to a logistic regression model to determine the sub-cellular localization of lncRNAs. Using the benchmark dataset, rich performance comparison with baseline machine learning models including Adaboost, Naive Bayes, Random Forest, Stacking classifier, and existing computational lncRNA sub-cellular localization predictors (Locate-R [208], lncLocator [206], iLoclncRNA [205]) indicated that lncLocPred attained promising performance. LncLocPred outperformed the state-of-the-art predictor by 2% on the benchmark dataset and 6% on the independent test set.

In Table 7, we have summarized state-of-the-art machine and deep learning based methodologies for the sub-cellular location prediction of coding and non-coding RNA.

### 3.4. Multi-Label Sub-Cellular Localization Prediction of Diverse RNAs

Biological functionalities of diverse biomolecules primarily rely on their distribution in cellular compartments. The presence of RNAs in different cellular compartments allows the cells to carry out a variety of biochemical processes concurrently. Taking the presence of RNAz into multiple compartments and the deficiency of a robust multi-label classification model capable of handling different RNAs into account, Wang et al. [210] extracted multi-label classification datasets related to sub-cellular localization of four different RNAs including miRNA, mRNA, lncRNA, and snoRNA. They evaluated six different nucleotide composition based encoding schemes including K-mer, RCKmer, NAC, DNC, TNC, and CKSNAP to efficiently capture the inherent relationships of the most discriminative sequence features. Multivariate information was fused using a multiple kernel learning paradigm based on a Hilbert–Schmidt independence criterion (HSIC), and optimal kernel combination was integrated with an SVM classifier to develop a robust multi-label predictor (MKSVM-HSIC) for the task of RNA sub-cellular localization. To prove the integrity of the proposed methodology MKSVM-HSIC, they compared the performance of MKSVM-HSIC with four other integration strategies using an SVM classifier such as binary relevance, label powerest, ensemble classifier, and multiple kernel learning using average weights (MK-AW). Empirical evaluation using four different RNA sub-cellular localization datasets indicated that the K-mer encoding scheme attained the best average precision of 0.68 for mRNA molecules and 0.745 for lncRNA molecules, whereas NAC achieved the peak average precision of 0.785 for miRNA molecules and DNC gained the top performance of 0.793 for snoRNA molecules. Among all integration strategies, multi-kernel SVM based on HISC achieved top average precision of 0.755, 0.754, 0.791, and 0.816 for mRNAs, lncRNAs, miRNAs, and snoRNAs followed by MK-AW (Table 7). To further illustrate the effectiveness of the proposed MKSVM-HSIC approach, authors compared the performance of MKSVM-HSIC with five different standalone classifiers including SVM, RF, ML-KNN, XGBT, and MLP. Performance evaluation of four RNA sub-cellular localization datasets once again proved the dominance of the proposed MKSVM-HSIC methodology which attained the top average precision of 0.703, 0.757, 0.787, and 0.800 on mRNAs, lncRNAs, miRNAs, and snoRNAs molecules, respectively (Table 7).

### 3.5. Benchmark Sub Cellular Localization Datasets

**Figure 4:**
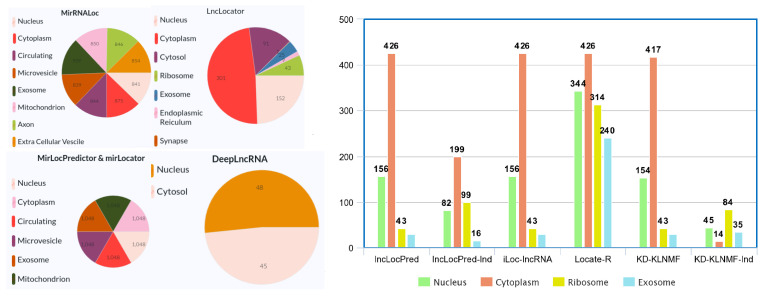
Statistics of different benchmark datasets used in the literature to evaluate the performance of ncRNA sub-cellular localization predictors.

In order to evaluate the integrity of diverse machine and deep learning approaches proposed for the determination of sub-cellular localization of different ncRNAs, a number of benchmark datasets have been developed. Researchers have mainly employed public metathesauruses such as RNA Central [169], RNAlocate [45], miRBase [44], and ENCODE [177] to develop unique sub-cellular localization information based datasets for ncRNAs. Figure 4 illustrates the statistics of different ncRNA sub-cellular localization datasets used in the literature.

Analyzing the first part of Figure 4 indicates that MiRNALoc [186] is evaluated on a benchmark dataset annotated against eight sub-cellular locations including Cytoplasm, Nucleus, Circulating, Microvesicle, Exosome, Mitochondrion, Axon, and Extracellular Vesicle, the sequence-subcellular location distribution of which is shown in the top leftmost pie chart (Figure 4). Performance of LncLocator [206] is assessed on a seven sub-cellular locations based dataset, the distribution of which is shown in the top rightmost pie chart (Figure 4). Both MirLocPredictor [187] and miRLocator [176] are evaluated on the same dataset comprised of six sub-cellular locations including Cytoplasm, Nucleus, Circulating, Microvesicle, Exosome, and Mitochondrion. DeepLncRNA [204] is evaluated on a small dataset annotated against only two sub-cellular locations, namely Cytosol and Nucleus.

Turning towards the second part of Figure 4, computational predictors including lncLocPred [209], iLoc-LncRNA [205], Locate-R [208], and KD-KLNMF [207] are evaluated on benchmark datasets annotated against four distinct sub-cellular locations such as Nucleus, Cytoplasm, Ribosome, and Exosome. Among all four approaches, the performance of two computational predictors lncLocPred [209] and KD-KLNMF [207] is additionally analyzed on the independent test set as well. For each dataset, the number of sequences against four different sub-cellular locations is depicted in the bar graph (Figure 4).

## 4. Current Challenges and Future Directions

Distinguishing ncRNAs from protein-coding transcripts and identifying their sub-type and sub-cellular localization patterns are the most important tasks to better understand the functionality, biogenesis, and complex mechanisms behind the development of distinct diseases and their potential to act as biomarkers. This paper sheds light on the progress of Artificial Intelligence related to identification of ncRNAs and their distribution patterns in cellular compartments. Unlike sub-cellular localization, a significant amount of work has been performed for the classification of RNA. We find that, in coding and ncRNA classification, state-of-the-art computational approaches mostly use alignment strategies. Furthermore, these methodologies utilize standard machine learning classifiers on manually extracted graph properties of RNA secondary structure. A critical analysis of diverse computational approaches indicates that pre-dominant lncRNA identification approaches make use of intrinsic sequence features such as ORF length, coverage, and integrity [58,59,60,61,77,78,83]. This is primarily because these features have good discriminative power as protein coding genes are ultimately transcribed as well as translated to yield a certain chain of amino acids that require unique nucleotide composition along with great quality open reading frames. Some predictive methodologies make use of transcript related features such as transcript length, higher order residues (K-mer), and composition–transcription–distribution [56,57,59,61,64,75,77,83,204] structure related features like molecular weight, Gravy, Instability [57,58,59,75,77,84], or protein-coding sequence features such as protein-coding sequence length [50,55]. However, few computational approaches utilize codon related features such as stop codon count, frequency [50,77], or GC content related features like frame score [51,77]. Existing approaches also do not investigate the potential of a wide range of feature selection approaches capable of eliminating redundant and irrelevant features. Furthermore, no researcher has attempted to explore the effectiveness of an attention mechanism that assigns higher weights to important features with the aim to improve the generalizability of deep neural networks. Despite considering the success of transfer learning, especially neural higher order embeddings for different genomic and bioinformatics tasks [211], no researcher has deeply investigated the performance of diverse types of neural higher order embeddings to learn rich inherent relationships of sequence residues. We consider comprehensive exploration of transfer learning, and bag of neural tricks has the potential to significantly increase the performance of lncRNA identification approaches.

For CircRNA classification, to date, six computational approaches have been developed where the majority of approaches utilize manually extracted features. In the quest for the development of a robust computational predictor which shall not rely on extensive pre-processing by making the best use of sequence information, more recent deep learning (CNN) based CircRNA classification methodologies managed to achieve the top performance of 98%.

Analysis of small ncRNA, especially piRNA identification approaches, reveals that, initially, the prime focus of researchers has been to develop one-layer computational predictors for identifying piRNA molecules. However, over the last few years, taking the biological importance of piRNA functionality into account, increasing the number of two-layer computational predictors are being developed to identify piRNA molecules and predict their core functionality. To date, a total of 18 computational predictors have been developed, out of which 14 predictors can be classified as one-layered approaches, whereas the remaining four predictors fall under the hood of two-layered approaches. For the first layer, piRNA predictors have managed to achieve the best performance of 98%, whereas, for the second layer, piRNA predictor best performance falls around 84%, indicating a lot of room for improvement.

It is quite evident from performance analysis that deep learning approaches perform better than machine learning approaches for small ncRNA classification and clustering tasks. However, there is still room for improvement, especially for the classification of small ncRNAs (e.g., piRNA function prediction). Although very deep neural architectures have more computational power, such architectures do not necessarily attain promising performance because they are prone to extracting irrelevant and redundant features. Generally, ncRNA sequence data (negative samples) including piRNA molecules contain many outliers and are highly sparse in nature; therefore, appropriate use of feature scaling, a balanced neural architecture with a suitable number of hidden layers, activation functions (e.g., sigmoid), and optimization function (e.g., Adam) found using neural architecture and hyper-parameter search algorithms (e.g., Particle SWARM) can increase the predictive performance up to a significant level.

In order to identify the family of small ncRNA sequences, to date, five computational predictors have been developed where predominantly secondary structure related features are used to represent small ncRNA sequences. Most of the computational predictors make use of deep neural networks; more specifically, recent DenseNet similar architecture achieved the best performance of 95% solely using raw sequences. This shows the potential of sequence information to accurately learn biological characteristics of sequence residues; therefore, more computational predictors on top of sequence information need to be developed to fill the performance gap.

Turning towards ncRNA sub-cellular localization, there exist several high-throughput controlled experimental approaches for the detection of RNA sub-cellular locations. However, limited computational approaches exist for RNA sub-cellular location prediction. RNAs mostly exist in more than one cellular compartment, which makes the identification of RNA locality at the cellular level a multi label classification task. Overall, in DNA and RNA sequence classification, most problems, such as coding and ncRNA classification, nucleuosome position detection, and histone markers identification, can be categorized as binary classification problems. Publicly available benchmark datasets of all these classification problems are almost balanced where positive and negative classes have an equal number of samples. Thus, for the aforementioned classification problems, computational approaches perform way better as they do not face data imbalance problems. On the other hand, for RNA sub-cellular location prediction, the deficiency of public annotated data sets is another major bottleneck. In addition, existing datasets have class imbalance problems and have a much lower number of samples for most classes. While computational approaches have achieved the performance of over 90% for lncRNA and mRNA molecules, miRNA sub-cellular localization performance is still around 60%. This is primarily due to less annotated data and failure of existing computational approaches to handle imbalanced sequences for multi-compartment distribution at different levels of the predictive pipeline. We consider that the use of multi-label data transformation approaches along with data over-sampling approaches (e.g SMOTE) have the potential to improve the predictive performance of miRNA sub-cellular localization.

## Figures and Tables

**Table 1:** A broad classification of computational approaches proposed to distinguish LncRNAs from protein-coding transcripts.

Method	Features	Alignment Approach	Feature Representation	Classification Paradigm	Algorithm	Source Code Availability	Database	Target Species	Performance			
									Acc	Spe	Sen	Auc
RNAz [53]	Thermodynamic stability measure, consensus secondary structure	pairwise and multiple sequence alignment	Z-Score			YES	Rfam	*E. coli*	0.7527	0.9893	0.7527	
CONC [54]	Amino acid composition, peptide length, predicted secondary structure content, predicted percentage of exposed residues, compositional entropy, number of homologs from database searches and alignment entropy	multiple sequence alignment	Protein properties of potential peptides from RNAs		SVM	NO	RNAdb, NONCODE, FANTOM	Eukaryotic		0.9520	0.9380	
				Binary Classifier								
PhyloCSF [66]	ORFs, Coding ECM	multiple alignments	probability of the alignment under the maximum likelihood estimate				fruitfly, Drosophila, melanogaster					
iSeeRNA [51]	conservation, ORF features sequence nucleotide composition	alignment-free	K-mer frequency			YES	RefSeq	Human, Mouse	0.96			0.98
CNCI [55]	nucleotide triplets	alignment-free	Usage Frequency of ANT			YES	GenCODE, Ensembl (v69)	Human, Plant	0.98			
PLEK [56]	Kmer features	alignment-free	Enhanced K-mer usage Frequency			YES	RefSeq, GenCODE	Human, Maize	0.956	0.955	0.925	
LncRScan-SVM [50]	transcript sequence, gene structure, codon sequence, conservation		Standard deviation of stop codon counts			YES	GENCODE	Human	0.922	0.953	0.891	0.966
CPC [57]	ORF, HIT SCORE	multiple alignments				NO	Rfam, RNAdb databank	Eukaryotic	0.932	0.873	0.995	
CPC2 [58]	Fickett score, ORF length, integrity, isoelectric point	CD-hit alignment	Combination of multiple features			YES	Rfam, RefSequence, Swiss-Prot	Human, Mouse, Fly, Zebrafish, Worm, Arabidopsis	0.96	0.97	0.95	
LongDist [49]	ORF absolute length, relative length occurrences of K-mers selected using principal component analysis		Combination of multiple features			YES	Ensembl	Human, Mouse, Zebrafish	0.982	0.974	0.989	
CPPred [59]	ORF length, integrity, coverage, hexamer score, Fickett score, pI, instability, gravy, composition- transition-distribution features		Combination of multiple features			YES	RefSeq	Human, Mouse	0.964	0.977	0.955	
LGC [52]	Relationship between ORF length and guanine-cytosine content	alignment-free	Combination of multiple features			YES	Ensembl, GENCODE	Human, Plant	0.945	0.925	0.964	
CPAT [60]	open reading frame size, open reading frame coverage, Fickett TESTCODE statistic, hexamer usage bias	alignment-free	Combination of multiple features		LR	YES	RefSeq, GenCODE	Human, Mouse, Fly, Zebrafish	0.966	0.97	0.96	0.9927
LncScore [61]	hexamer score, ORF length, coverage, Hexamer score distance, maximum coding subsequences, Fickett score	alignment-free	Combination of multiple features			YES	GENCODE	Human, Mouse	0.964	0.940	0.973	0.994

**Table 2:** A Broad Classification of Approaches Proposed to Distinguish LncRNAs from Protein-Coding Transcripts.

Method	Features	Alignment Approach	Feature Representation	Classifier Type	Algorithm	Source Code Availability	Database	Target Species	Performance			
									Acc	Spe	Sen	Auc
LncRNA-ID [62]	open reading frame (ORF), protein conservation and ribosome interaction	profile hidden Markov model (profile HMM)-based alignment	Combination of multiple features		RF	YES	LncRNA Disease database	Human, Mouse	0.9578	0.9528	0.9628	
LncRNApred [69]	MaxORF, RMaxORF and SNR		self organizing feature map	Machine Learning		NO	UCSC, NONCODE	Human	0.929	0.925	0.934	0.973
FEELnc [64]	open reading frame, K-mer frequencies	alignment-free	Combination of multiple features			YES	NON-CODE, GENCODE	Human, Mouse, Dog	0.939	0.941	0.938	
COME [63]	GC content DNA conservation Protein conservation poly(A)- expression poly(A)+ expression small RNA expression H3K36me3 H3K4me3 RNA structure conservation	multiple alignments	Combination of multiple features			YES	lncRNAdb, RefSeq	Human, Mouse, Fly, Worm, Plant	0.947	0.963	0.897	0.981
LncFinder [75]	Sequence intrinsic features, physicochemical property based features, and secondary structure features	alignment-free	Combination of multiple features	Machine Learning	Meta-Classifier	YES	GENCODE	Human, Mouse, Wheat, Zebrafish, Chicken	0.974	0.973	0.964	0.991
PredLnc-GFStack [77]	codon-related features, ORF-related features GC-related features coding sequence-related features transcript-related features structure-related features	CD-HIT alignment	Combination of multiple features			YES	GENCODE	human, mouse, zebrafish, fruit fly, *S. cerevisiae*, nematode thale cress	0.914	0.933	0.875	0.969
LncPred-IEL [76]	ORF length, coverage, integrity, Fickett score, hexamer score, gravy, instability, Spectrum, composition–transition–distribution, mismatch, reverse compliment K-mer, pseudo nucleotide composition, and auto-cross variance features	CD-HIT alignment	Combination of multiple features			YES	GENCODE	Human, Mouse, Fruitfly, Zebrafish	0.959	0.976	0.856	0.984
CPE-SLDI [78]	ORF length, coverage, integrity, Fickett score, hexamer score, gravy, instability, composition–transition–distribution		Combination of multiple features			YES	GENCODE	Human, Mouse, Human-sORF, Mouse-sORF	0.97			
IncRNA-MFDL [80]	ORF, secondary structure, most like coding domain transcript, K-mer		Combination of multiple features	Deep Learning	DSNN	YES	GENCODE, RefSeq	Human, Anole lizard, Zebrafish, Chicken, Gorilla, Macaque, Mouse, Lamprey, Orangutan, *Xenopus*, and *C. elegans*	0.971	0.965	0.977	
DeepLnc [81]	k-mer frequencies	Shannon Entropy feature based alignment	Combination of multiple features		DNN	NO	LNCipedia, RefSeq	Human	0.980	0.971	0.989	0.993
mRNN [48]	K-mers		One-hot encoding		RNN	YES	GENCODE	Human, Mouse	0.98	0.999	0.971	0.984
LncRNANet [84]	Open reading frame (ORF) indicator	multiple sequence alignment	One-hot encoding		RNN, CNN	YES	GENCODE, ENSEMBL and Human and Vertebrate Analysis and Annotation group databases	Human, Mouse	0.9179	0.8766	0.9591	
LncADeep [83]	ORF length, hexamer score, Fickett score		Combination of multiple features		DBN, DNN	YES	GENCODE, RefSeq	Human, Mouse		0.972	0.981	
DangCNN [85]	k-mer occurrence matrix		K-mer frequency		CNN	YES	GENCODE	Human, Mice, Chicken			0.995	1.00

**Table 3:** A broad classification of computational approaches proposed to discriminate CircRNAs from other ncRNAs.

Method	Features	Redundancy Removal Approach	Feature Representation	Classification Task Type	Algorithm	Source Code/ Web Server Availbility	Database	Target Species	Performance				
									ACC	SEN	SPE	PRE	MCC
PredcircRNA [94]	graph features, sequence composition, conservation information, tandem repeat, ALU, ORF features, SNP density	RT-PCR, HAVANA manual annotation	fusion of heterogeneous features	Multi -Class	MKL, RF, SVM	YES	Circbase, GENCODE, circRNADb	Human	0.862	0.864	0.859	0.865	0.724
H-ELM [95]	graph features, conservation score features, component composition features, ALU, tandem repeats, ORF, SNPs	RT-PCR, HAVANA manual annotation	MMR and IFS based fusion of features		hierarchical extreme learning machine	YES	Circbase, GENCODE, circRNADb	Human	0.789	0.703	0.850		0.561
CircCode [96]		Filtering of ribosomal profiling data			ML Classifier	YES	NCBI, Ensembl, RPFdb, CIRCPEDIA, PlantcircBase	Human, Arabidopsis Thaliana	0.60				
CirRNAPL [97]	Ribonucleic acid composition, Autocorrelation, Pseudo- ribonucleic acid composition, Predicted structure composition	Eliminating Overlapping and Short Sequences (<200 nt)	Fusion of multiple features		extreme learning machine based on particle swarm optimization algorithm	YES	Circbase, GENCODE, circRNADb	Human lncRNAs, PCTS, Stem Cells	0.815	0.802	0.795		0.635
CircDeep [98]	Manually Curated Features, K-mer Features	Eliminating Short Sequences (<200 nt)	Fusion of reverse complement matching, conservation descriptor, and ACNN-BLSTM Features		ACNN-BiLSTM	YES	Circbase, GENCODE, circRNADb	Human	0.949	0.955	0.938		0.845
CircNet	Sequence K-mer features	Eliminating Short Sequences (<200 nt)	CNN-Autoencoder		CNN	No	Circbase, GENCODE, circRNADb	Human	0.9828		0.9775		0.9635

**Table 4:** A broad classification of computational approaches proposed to identify piRNA molecules.

Method	Features	Redundancy Removal Approach	Feature Representation	1-Layer/2-Layer	Classification Paradigm	Algorithm	Source Code /Web Server Availbility	Database	Target Species	Performance				
										ACC	SEN	SPE	PRE	MCC
BetelPredictor [114]	Sequence Features	WU-BLAST and Newly Developed Tool	position specific residues properties	One Layer		SVM	NO		Mouse				0.61	
piRNAPredictor [115]	K-mer features		K-mer based representation	One Layer		SVM	YES	NONCODE, NCBI	rat, mouse, human, fruit fly, nematode.		0.60		0.90	
Piano [116]	Triplet elements combining structure and sequence information	SeqMap		One Layer		SVM	NO	GenBank, NONCODE, UCSC	human, mouse, rat, Drosophila	0.95	0.96	0.9461	0.9495	
PiRPred [117]	K-mer		K-mer based representation	One Layer		Multi-Kernel SVM	YES	GtRNAdb, Biomart	human, Drosophila	0.89	0.83	0.95		
Pibomd [118]	Sequence Motifs			One Layer		SVM	YES	NONCODE, NCBI	rat, mouse, human, fruit fly, nematode	0.906	0.915	0.898		
McRUM [119]	correlation based K-mer features		K-mer based representation	One Layer		L1 based SVM	NO	NONCODE, NCBI	*Caenorhabditis elegans*, Human, *Locusta migratoria*, *Drosophila melanogaster*	0.931	0.939	0.923		0.862
LiuPredictor [120]	weighted K-mer features		K-mer based representation	One Layer		SVM	NO	NONCODE, NCBI	human, mouse, drosophila, and rat		0.90		0.90	
2L-piRNA [121]	physicochemical properties of nucleotides pseudo K-tuple nucleotide composition	CD-Hit	multiple features	Two Layer		SVM	YES	piRBASE, NONCODE	*M. musculus*	86.1, 0.776	88.3, 0.791	83.9, 0.76		0.723, 0.552
piRNAdetect [122]	n-gram model based features			One Layer		SVM	NO	piRBASE	*H. sapiens* *R. norvegicus* *M. musculus*	0.844				
piRNApred [123]	physico-chemical properties based features	CD-Hit		One Layer		SVM	NO	piRBASE, NONCODE	*H. sapiens*, *M. musculus*, *D. melanogaster*, *C. elegans* *Danio rerio*, *Gallus gallus domesticus*, *Xenopus tropicalus*, *Bombyx mori*	0.986	0.986	0.986		0.97
IpiRId [124]	K-mer motifs features	BLAST		One Layer		SVM	YES	piRBASE, piRNABank, GtRNAdb,	Human, Mouse, Fly	0.936	0.907	0.966	0.964	
2L-piRNAPred [125]	single, dinucleotides composition, physicochemical properties, position specificities of nucleotides, proportions of peptide sequence	CD-Hit	F-score based fusion of multiple features	Two Layer	Machine Learning	SVM	NO	piRBASE, NONCODE	*M. musculus*	0.89, 0.84	0.904, 0.843	0.875, 0.836		0.779, 0.68
2lpiRNApred [126]	K-mer, General parallel correlation pseudo-dinucleotide composition, General series correlation pseudo-dinucleotide composition, Normalized Moreau–Broto autocorrelation, and Geary autocorrelation	CD-Hit		Two Layer		Sparse Representation, SVM classifier	YES	piRBASE, NONCODE	*M. musculus*	0.887, 0.806	0.919, 0.824	0.855, 0.776		0.776, 0.600
GA-WE [127]	multiple K-mer related features	SeqMap		One Layer		Weighted Random Forest	YES	NONCODE, NCBI, UCSC	Human, mouse, Drosophila	0.964	0.940	0.973		0.694
LuoPredictor [128]	Physico-chemical Properties based features	SeqMap		One Layer	Machine Learning	Random Forest	YES	NONCODE UCSC	Human, Mouse and Drosophila	0.958	0.952	0.965		
ine V-ELMpiRNAPred [129]	short sequence motifs with K-mer features			One Layer		Voting based Extreme Learning Machine	NO	NONCODE	Human	0.952	0.956	0.947		0.899
piRNN [130]	K-mer features			One Layer	Deep Learning	CNN	YES	miRBASE, tRNA database	*Caenorhabditis elegans*, *Drosophila melanogaster*, rat and human	0.95	0.97	0.97	0.94	0.91
2L-piRNADNN [131]	di-nucleotide auto covariance features with 6 physico-chemical properties based features			Two Layer		DNN	YES	piRBASE, NONCODE	*M. musculus*	0.918, 0.845	0.909, 0.812	0.948, 0.903		0.821, 0.650

**Table 5:** Summary of machine and deep learning based methodologies proposed for the identification and clustering of non-coding RNAs.

Method	Database	Alignment Features	Features	Classification Approach	Target Biomoelcule and Sequence Analysis Task	Performance			
						Accuracy	Specificity	Sensitivity	AUC
Deep RNN [149]	NCBI, fRNAdb, NON-CODE	pairwise sequence alignment	Secondary sequence features	RNN	Micro RNAs Identification	-	0.9920	0.8220	-
EnsembleClust [150]	ENSEMBL	Pairwise sequence alignment	structural alignments score	Hierarchical Clustering	Clustering of non coding RNA	-	-	-	0.944
RNAscCLust [151]	Rfam	-	structure conservation and graph-based motifs	Hierarchical Clustering	Clustering of non coding RNA	-	-	-	-
SHARAKU [152]	NCBI Reference sequence database, ENSEMBL database and next generation sequencing output	Pairwise sequence alignment	Similarity score matrix	Random forest	Clustering of non coding RNA	-	-	-	0.985
CNNClust [153]	Rfam, HUGO gene nomenclature committee (HGNC) databases, Ensembl and genomic tRNA database	Pairwise sequence alignment	Derived position weight matrices of sequence motifs	CNN	Clustering of non coding RNA	0.9800	-	-	-

**Table 6:** A brief summary of computational methodologies developed for small non-coding RNA family classification.

Method		Database	Alignment Features	Features	Classification Approach	Target Biomoelcule	Performance			
							ACC	SPE	SEN	AU-ROC
Hybrid Random Forest [160]		Rfam, RefSeq, NCBI GenBank, genome database, lncRNAdb database	Multiple sequence alignment	sequence, structure, structural robustness, modularity and coding potential	Random Forest	Classification into small non coding or long non coding RNA	0.9211	0.9350	0.9070	
ine	Deep next generation sequencing [163]	NONCODE, NCBI, lncRNA	Pairwise sequence alignment	Protein coding features	Deep next generation sequencing	Classification into coding or non coding RNA	-	-	-	-
ine nRC [164]		Rfam	Multiple sequence alignment	Secondary structure features	CNN	Classification of small non coding RNA	0.8181	0.9848	0.8181	-
ine RNAGCN [161]		Rfam	Multiple sequence alignment	Secondary structure features	graph convolutional network	Classification of small non coding RNA	0.8573	-	-	-
ine RPC-snRC [47]		Rfam	-	Raw Sequence	Dense-Net	Classification of small non coding RNA	0.9538	-	-	-

**Table 7:** Summary of machine and deep learning based methodologies for the sub-cellular location prediction of coding and non-coding RNA.

Method	Database	Features	Classification Approach	Target Biomolecule	Performance
miRGOFS [185]	RNALocate [45]	Correlation scores	SVM	miRNA	F1 Score: 0.612
MiRLocator [176]	RNALocate [45]	Raw Sequence	sequence to sequence model	miRNA	F1 Score: 0.4933
miRNALoc [186]	RNALocate [45], miRBase [44]	pseudo di-nucleotides compositions, thermodynamic, and physico-chemical properties	SVM	miRNA	AU-ROC Score: 63–71%
MirLocPredictor [187]	RNALocate [45]	kmerPR2Vec Features	CNN	miRNA	F1 Score: 0.6178
RNATracker [178]	Ensembl database [46]	secondary structure information,	CNN, LSTM	mRNA	Pearson: 0.604
iLoc-mRNA [179]	GENBANK, RNALocate databases [45]	binomial distribution and variance analysis,	SVM	mRNA	accuracy: 0.9012
mRNALoc [180]	GENBANK, RNALocate databases [45]	pseudo K-tuple nucleotide composition,	SVM	mRNA	Jackknife accuracy: 0.99
DM3LOC [181]	GenBank and RNALocate databases [45]	one-hot encoding sequence information,	multi-head self attention CNN	mRNA	Average AU-ROC: 0.7416
DeepLncRNA [204]	ENCODE project [177]	Raw Sequence	Deep Neural Network	lncRNA	Accuracy: 0.724
iLoc-lncRNA [205]	RNALocate [45]	Pseudo K-tuple Nucleotide Composition	SVM	lncRNA	Accuracy: 0.8672
lncLocator [206]	RNAlocate [45]	K-mer nucleotide composition features	ensemble classifier	lncRNA	Accuracy: 0.598
KD-KLNMF [207]	RNAlocate [45]	Dinucleotide based spatial autocorrelation, k-mer descriptors, nonnegative matrix factorization	SVM	lncRNA	Accuracy: 0.97
Locate-R [208]	RNAlocate [45]	n-gaped l-mers	SVM	lncRNA	Accuracy: 0.89
lncLocPred [209]	RNAlocate [45]	K-mer, Pseudo Dinucleotide Composition, Local Structure–Sequence Triplet Element AND binomial distribution, variance threshold, F-score	Logistic Regression	lncRNA	Accuracy: 0.91
MKSVM-HSIC [210]	RNAlocate [45]	K-mer, CKSNAP, DNC, TNC, NAC, RCKmer	Multi-Kernel learning based SVM	mRNA, lncRNA, miRNA, snoRNA	Average Precision: 0.755, 0.754, 0.791, 0.816

## Data Availability

All relevant data is contained within the article.
